# Expression of TMBIM6 in Cancers: The Involvement of Sp1 and PKC

**DOI:** 10.3390/cancers11070974

**Published:** 2019-07-11

**Authors:** Raghu Patil Junjappa, Hyun-Kyoung Kim, Seong Yeol Park, Kashi Raj Bhattarai, Kyung-Woon Kim, Jae-Won Soh, Hyung-Ryong Kim, Han-Jung Chae

**Affiliations:** 1 Department of Pharmacology and New Drug Development Research Institute, Chonbuk National University Medical School, Jeonju 54896, Korea; 2 Animal Biotechnology Division, National Institute of Animal Science, Rural Development Administration (RDA), Wanju-gun, Chonbuk 54875, Korea; 3 Department of Chemistry, Inha University, Incheon 402-751, Korea; 4 College of Dentistry, Institute of Tissue Regeneration Engineering (ITREN), Dankook University, Cheonan 31116, Korea

**Keywords:** TMBIM6, promoter, transcriptional regulation, Sp1, PKC, cancer

## Abstract

Transmembrane Bax Inhibitor Motif-containing 6 (TMBIM6) is upregulated in several cancer types and involved in the metastasis. Specific downregulation of TMBIM6 results in cancer cell death. However, the TMBIM6 gene transcriptional regulation in normal and cancer cells is least studied. Here, we identified the core promoter region (−133/+30 bp) sufficient for promoter activity of TMBIM6 gene. Reporter gene expression with mutations at transcription factor binding sites, EMSA, supershift, and ChIP assays demonstrated that Sp1 is an essential transcription factor for basal promoter activity of TMBIM6. The TMBIM6 mRNA expression was increased with Sp1 levels in a concentration dependent manner. Ablation of Sp1 through siRNA or inhibition with mithramycin-A reduced the TMBIM6 mRNA expression. We also found that the protein kinase-C activation stimulates promoter activity and endogenous TMBIM6 mRNA by 2- to 2.5-fold. Additionally, overexpression of active mutants of PKCι, PKCε, and PKCδ increased TMBIM6 expression by enhancing nuclear translocation of Sp1. Immunohistochemistry analyses confirmed that the expression levels of PKCι, Sp1, and TMBIM6 were correlated with one another in samples from human breast, prostate, and liver cancer patients. Altogether, this study suggests the involvement of Sp1 in basal transcription and PKC in the enhanced expression of TMBIM6 in cancer.

## 1. Introduction

Transmembrane Bax Inhibitor Motif-containing 6 (TMBIM6), is also known as Bax Inhibitor-1, is an evolutionarily conserved transmembrane protein predominantly localized in the endoplasmic reticulum (ER) membrane [1]. The TMBIM6 gene was first identified while screening the testis cDNA library and was thus named the testis enhanced gene transcript [2]. Later, while screening cDNA expression libraries for clones that protect yeast from Bax-mediated cell death, TMBIM6 was identified as an antiapoptotic protein that inhibits Bax-mediated cell death [3,4]. The human TMBIM6 gene is located on chromosome 12q13.12 and expressed in two transcriptional variant isoforms [2].

TMBIM6 gene is normally expressed in all tissues but the expression level varies depending on the tissue [5]. However, the dysregulated expression of TMBIM6 leads to cell transformation, which can result in tumorigenicity [6,7]. High expression of TMBIM6 has been observed in many cancer types, such as breast, prostate, non-small cell lung, nasopharyngeal, and pulmonary adenocarcinoma [8,9,10,11,12,13]. A high level of TMBIM6 expression mediates resistance to apoptosis in carcinoma cells [14]. Additionally, TMBIM6 overexpression promotes cancer metastasis by regulating the actin polymerization, glucose metabolism, and mitochondrial function [15]. Specific suppression of TMBIM6 expression through RNA interference leads to the cell death and reduced tumor development in nasopharyngeal carcinoma [14], prostate carcinoma cells [10], and breast cancer cells [9]. Increased expression of TMBIM6 in some cancers is not due to any mutations in the upstream or coding region, rather it resulted from transcriptional regulatory elements in the tumor environment [8].

Apart from its pathological relevance, TMBIM6 is involved in many physiological processes, such as maintaining calcium homeostasis [16,17]; ROS regulation [18]; adaptive immune response [19]; and cell proliferation, apoptosis, and differentiation [20]. Increasing evidences supporting the involvement of TMBIM6 in physiological processes and in various cancer developments led us to study the transcriptional regulation of TMBIM6, which has not been studied much. Understanding the basal and enhanced transcriptional regulation of the human TMBIM6 gene could be useful in identifying innovative strategies for specific cancer therapies and regulating TMBIM6 expression to protect against cellular injury.

On this background, we selected the 5′ flanking region of ubiquitously expressed human TMBIM6 transcript isoform-1 for this investigation. We looked for the molecular mechanisms of gene regulation by identifying the cis-regulatory elements and analyzing their activities in different carcinoma cell lines. Furthermore, this study explored the significance of the Sp1 and PKC in the upregulation of TMBIM6 expression in cancers.

## 2. Results

### 2.1. Mapping of Transcription Start Site (TSS) of Human TMBIM6 Gene

**Figure 1:**
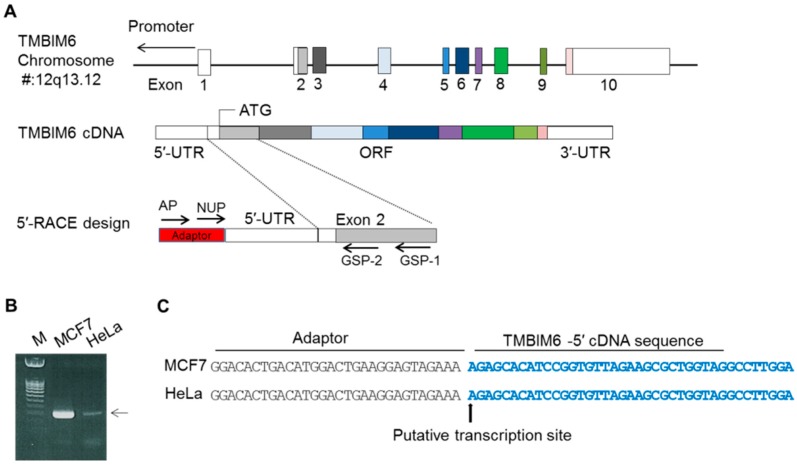
Identification of the transcription start site (TSS) of the human TMBIM6 gene. (**A**) Schematic representation of the genome span (23.5 kb) of the TMBIM6 gene in chromosome 12q13.12. The box represents the exon positions between introns; empty box represents the UTR regions; color shaded box shows the ORF. The color box line shows the TMBIM6 cDNA with the ATG site and 10 exons. Next, 5′-RACE PCR, with adapter and nested PCR joined to the 5′ end of the cDNA and gene-specific primers GSP1 and GSP2 specific to exon 2, full length cDNA was amplified. (**B**) 5′-RACE PCR products were separated by gel electrophoresis. (**C**) Sequence analysis after the PCR product clones; the arrow shows the position of the TSS (+1) of the TMBIM6 gene.

We first looked into the GenBank and UCSC genome browsers for available information about human TMBIM6 cDNA. Two transcription variants have been reported for human TMBIM6 (NM_003217.3 and NM_001098576.1). As mentioned in a previous study [21], in rats, two TMBIM6 transcriptional variants have been found: variant-1 is from the distal promoter P2, which is expressed ubiquitously; and variant-2 is from the proximal promoter P1, which is restricted to the testes. We tested the expression pattern of both variants of human TMBIM6 in cells other than testis tissue by RT-PCR using exon-1-specific forward primers and common exon 2 primers, and we found that only variant-1 is expressed in HT1080 and HeLa cells (Supplementary Figure S1A). This study focused on characterizing the transcriptional regulation of ubiquitously expressed human TMBIM6 variant-1 (NM_003217.3). Based on the information available in the UCSC genome browser, mapping of human TMBIM6 transcriptional variant-1 was done. Its total genomic span is ~23.5 kb in chromosome 12q13.12, and it has a total of 10 exons (Figure 1A).

As a primary step, we identified the TMBIM6 transcription start site (TSS) using a 5′-RACE PCR. This technique enables the amplification of cDNA from only the full length, 5′-capped mRNA, excluding the amplification of incomplete cDNA. Therefore, the identified 5′-cDNA end represents the TSS of the gene. The 5′-RACE analysis was performed using reverse oligonucleotides (GSP1 and GSP2) specific to exon-2 of the coding region and two adapter primers for the primary and nested PCR (Figure 1A). To find the TSS site, cell lines were selected based on the endogenous TMBIM6 expression (Supplementary Figure S1B). Among tested cell lines, MCF7 (expressing a high level of TMBIM6) and HeLa (expressing low levels of TMBIM6), were used for the TSS analysis. cDNA was synthesized from the total RNA extracts of MCF-7 and HeLa cells, and the first PCR amplification was performed with outer primer AP1 and reverse primer GSP1. Those first PCR products were then used as a template for the nested PCR with NUP and GSP2 primers (Figure 1B). The 5′-RACE products of the two cells were gel-purified and cloned into the pGEMT-easy vector. A DNA sequence analysis from 10 PCR clones showed that the sequence obtained is identical to the 5′-end of the TMBIM6 sequence available in GenBank (NM_003217.3); no new sequence was found. The TSS is 85 bp upstream from the translation start site (ATG). In the further experiments, the 5′-end nucleotide ‘A’ was defined as the TSS and numbered as +1.

### 2.2. Identification of the Proximal Promoter Region of TMBIM6

**Figure 2:**
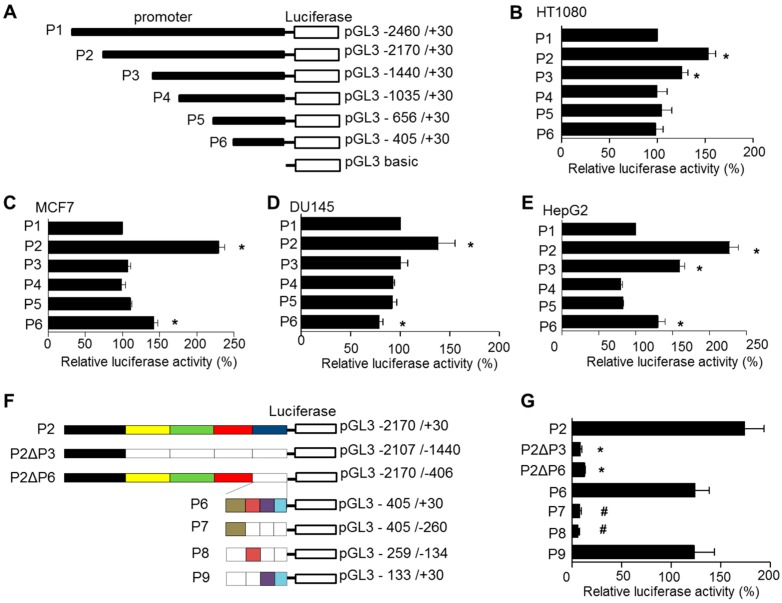
Characterization of the human TMBIM6 promoter. (**A**) Schematic presentation of a series of 5′ flanking region deletion constructs between −2460 and +30 of TMBIM6. Each construct was made in the pGL3-basic promoter-less luciferase reporter plasmid. Constructs were named P1–P6, and the nucleotide numbers of each construct are relative to the TSS. (**B**–**E**) Each construct was transfected into HT1080 (**B**), MCF-7 (**C**), Du145 (**D**), and HepG2 (**E**) cells, and the luciferase activity was measured after 24 h and normalized to β-gal activity to account for transfection efficiency. The luciferase activity of each construct is presented relative to the activity of the pGL3-basic vector. The results are the mean ± SD from *n* = 3 independent experiments. ‘*’ indicates significant differences from the P1. (**F**) Show the design of the P2 deletion constructs and fragmented P6 constructs in the pGL3-basic reporter vector. Empty boxes represent the deleted positions. The nucleotide positions of each construct are relative to the TSS. (**G**) Relative luciferase activity of each truncated P2 constructs and fragmented P6 constructs P7, P8 and P9; P9 construct is identified as the core promoter of TMBIM6. The results are the mean ± SD from *n* = 3 independent experiments. ‘*’ indicates significant differences from the P2. ‘#’ indicates significant differences from P6.

To find the minimal promoter region that regulates TMBIM6 gene expression, we cloned the 5′-flanking region upstream from the TSS. Total genomic DNA was extracted from fibrosarcoma HT1080 cells and used as a template for cloning the 5′-flanking region, using the upstream TMBIM6 genomic sequence information in the UCSC genome browser. A total of 2460 bp (−2430 to +30) (Supplementary Figure S2) were cloned into the promoter-less pGL3 basic vector to generate reporter constructs. Then, we generated a series of 5′ end deletion constructs (P1 to P6) containing specified genomic fragments of the TMBIM6 gene (Figure 2A). To determine which reporter construct is involved in transcriptional regulation, each reporter construct was transfected into four cell lines, representing different tissues, as HT1080, HepG2, MCF7, and DU145, and luciferase activity was measured after 24 h. All six reporter constructs showed similar luciferase activity except P2 (−2170/+30), which showed very high activity irrespective of the cell line tested (Figure 2B–E). Progressive deletion of sequences down to P6 (−435/+30) did not alter the activity significantly when compared with the longest construct (P1, −2430/+30). P6 consistently showed considerable promoter activity in all cell lines tested. Therefore, it was presumed that the smaller construct P6 might contain the elements necessary for basal promoter activity. However, the P2 construct also showed the maximum promoter activity, indicating its contribution to the TMBIM6 gene expression. To rule out this dilemma, we cloned a P2-specific region (P2ΔP3) excluding P3 and in another clone, deleted the P6-specific region from the P2 construct (P2ΔP6) (Figure 2F). Surprisingly, P2ΔP3 and P2ΔP6, deletion of the P6-specific region from P2 resulted in a complete loss of activity (Figure 2G). These results suggested that the P6 region contains necessary elements which are sufficient for basal transcriptional regulation of TMBIM6 gene.

To further characterize the P6 reporter construct HT1080 cells were used, as all P1-P6 constructs showed high levels of promoter activity compared to other tested cell lines (Figure 2B–E).We have fragmented P6 into three regions by PCR amplification and cloned those fragments into reporter vector constructs named P7 (−405/−260), P8 (−259/−134), and P9 (−133/+30) (Figure 2F). Those constructs were transfected into HT1080 cells to measure luciferase activity. We found that P7 and P8 showed no activity, whereas P9 showed activity similar to that seen with P6 (Figure 2G). Therefore, we consider P9 to be the minimal proximal promoter; it contains nucleotides from −133 bp to +30 bp, which have positive regulatory elements, and it is sufficient to drive the basal promoter activity for TMBIM6 gene.

### 2.3. Analysis of the Proximal Promoter of the TMBIM6 Gene for Potential Interacting Factors

**Figure 3:**
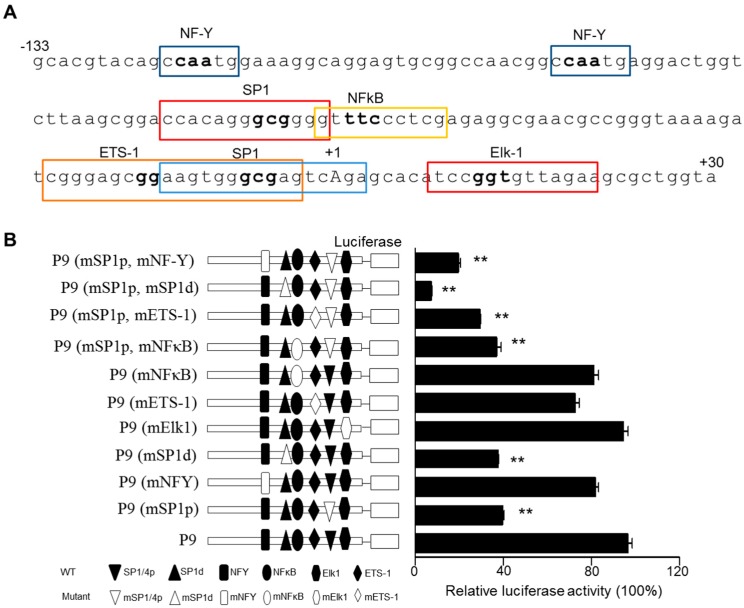
Two putative Sp1 sites are required for the basal promoter activity of human TMBIM6. (**A**) Nucleotide sequence of the core promoter region (−133/+30) of TMBIM6, with putative transcription factor binding sites identified in the MatInspector program, have a sequence similarity index >0.9. Highlighted nucleotides are selected for site-directed mutagenesis. The TSS is designated as +1. (**B**) Site-directed mutated constructs of identified transcription factor site; each mutation was pictorially represented in comparison to the P9 mother construct. Each mutated construct was transiently transfected into HT1080 cells. After 24 h, luciferase activity was measured. The results are the mean ± SD from *n* = 3 independent experiments. ‘**’ indicates significant differences from the P9 at *p* < 0.01.

The minimal promoter region P9, covering −133 bp to +30 bp, was analyzed for putative transcription factor binding sites using MatInspector professional (http://www.genomatix.de/) and TFSEARCH software. Transcriptional factor binding sites with a high matrix similarity index (>0.9) were considered. First, we found no TATA box presence in the selected TMBIM6 promoter region, which is commonly located between 25 and 35 bp upstream of the TSS [22]. Figure 3A shows representative putative transcription sites with high matrix similarity, which is necessary for a TATA-box-less promoter. The analysis showed that minimal promoter region P9 contains sites for ubiquitous factors: two Sp1, two NF-Y (CAAT box), one each of ETS-1, Elk-1, and NF-κB. If two sites were found for a single factor, we named them as proximal and distal sites based on their relative distance from the TSS. As a point of interest, P1 specific and P2 specific regions were also analyzed for potential transcription factor binding sites, in which no Sp1 binding sites, but other putative sites such as NF-Y and ETS-1 were observed (Supplementary Figure S3).

To understand the significance of these putative transcription sites, we introduced two or three nucleotide substitutions using mutations of nucleotides within the matrix of element binding sites (based on MatInspector data). The site-specific mutated constructs in HT1080 cells were tested for luciferase activity after 24 h transfection, along with the parent P9 construct. The results demonstrated that a single mutation in the proximal Sp1 and distal Sp1 individually caused a significant reduction (~60–70%) in activity, and a double mutation of both Sp1 sites resulted in a 90% reduction in luciferase activity (Figure 3B). Mutation of other sites, including the CAAT box site, did not significantly affect the functional activity (~10% reduction) of the P9 construct (Figure 3B). Furthermore, double mutations of the proximal Sp1 site plus the CAAT box, ETS1and NF-κB caused 80%, 70%, and 60% reductions, respectively. These results strongly suggest that the Sp1 factor is critical to maintaining the basal promoter activity of TMBIM6. The other factors could influence the basal function, but individually, they do not control the promoter activity. Therefore, we further focused on the Sp1 transcription factor. The sequence alignment of 150 bp upstream from the TSS of TMBIM6 gene in human, rat, and mouse DNA revealed that the Sp1 region is highly conserved across species (Supplementary Figure S4).

### 2.4. Sp1 Binds to the TMBIM6 Promoter: EMSA and ChIP Assay

**Figure 4:**
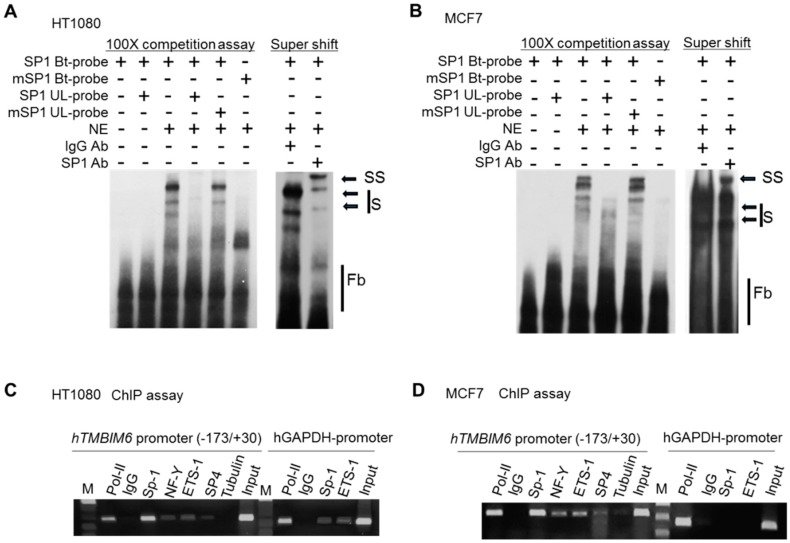
EMSA and ChIP assay showing Sp1 binding to the human TMBIM6 promoter. (**A**,**B**) Nuclear extract (NE) was prepared from HT1080 (A) and MCF7 (B) cells. Double-stranded 35-bp oligonucleotide probe specific to Sp1 proximal site (−25/+7) was generated and labeled with biotin. The labeled Sp1-p probe was incubated alone (lane 1), with unlabeled probe (lane 2), with the NE (lane 3), with the NE and 200-fold excess amount of unlabeled homologous probe (lane 4), with 200-fold excess mutant probe (lane 5), and with mutant-labeled probe that failed to form a complex with the NE (lane 6). DNA–protein complexes that formed are indicated (S), unbound probes indicated as free probes (Fb). In lanes 7–9, the super shift assay was performed by pre-incubating the NE with probe for 20 mins on ice before adding the specific antibody. Then, the specific probe was added and further incubated for 20 mins on ice, with normal IgG as the negative control in lane 7 and anti-Sp1 monoclonal antibody in lanes 8, respectively. The super shift is indicated as SS. (**C**,**D**) ChIP was carried out with HT1080 (**C**) and MCF7 (**D**) cells; antibodies for RNA polymerase II (pol-II), Sp1, ETS-1, NF-Y, and SP4 were used, with tubulin and IgG as the negative control. Immunoprecipitated chromatin was amplified by PCR with primers specific to region −173 to +30 of the TMBIM6 gene. The GAPDH locus was used as the control. M: DNA marker.

An EMSA assay was carried out to confirm the actual binding of the putative transcription factors identified during the mutation studies. Double-stranded DNA probes of 35 bp corresponding to the two Sp1 sites in the P9 promoter region of TMBIM6 were 3′ end-labeled with biotin. Then EMSA was performed with the nuclear extract of HT1080 and MCF7 cells. Figure 4A displays that the distal Sp1 site (−68/−51) in the wild type probe binds to the nuclear extract (lane 3) of HT1080 cells. Multiple shifted bands were observed, indicating the formation of multiple DNA probe–nuclear protein complexes. However, those bands competed efficiently with a 100-fold molar excess of unlabeled homologous probe (Figure 4A, lane 4), but not with the same probe with a four-nucleotide mutation at the Sp1 site (lane 5). Similarly, the homologous labeled mutant probes did not bind to the nuclear extract (Figure 4A, lane 6). The addition of Sp1-specific antibody to the reaction super-shifted the probes (Figure 4A, lane 8) but the negative control IgG failed to shift the probes (lane 7). Similar results were observed with the nuclear extract of MCF7 cells (Figure 4B). Proximal Sp1 site (−15/+3) specific probes also showed similar results (Supplementary Figure S5). These results show that lysates from different tissue contain Sp1 that interacts with the sequences of the TMBIM6 promoter comprising the proximal and distal Sp1 binding sites. These observations infer that both proximal and distal sites bind Sp1.

To further confirm the binding of the Sp1 factor to the TMBIM6 promoter region in living cells, we conducted a ChIP assay. Cross-linked chromatin from HT1080 and MCF-7 cells were immunoprecipitated with anti-Sp1, anti-ETS-1, anti-SP4, and anti-NF-Y (CAAT box) monoclonal antibodies individually. Then, the TMBIM6 5′-flanking region was amplified from the ChIP precipitated DNA by PCR (sequence between −200/+30). The results confirmed that the endogenous Sp1 protein is prominently bound to the TMBIM6 promoter region in cultured HT1080 cells, whereas the other proteins showed less association (Figure 4C). Furthermore, the interaction of the above-mentioned factors with TMBIM6 was assayed in MCF-7 cells, but those results showed that increased binding of ETS-1 and NF-Y are observed compared to HT1080 cells (Figure 4D). However, in both cell lines tested, Sp1 showed the prominent binding with TMBIM6 promoter region. These results are consistent with the cell-free EMSA/super shift assays (Figure 4A,B). All these observations support the major involvement of Sp1 in TMBIM6 transcriptional regulation and other factors such as ETS-1, NF-Y may corroborate the Sp1-mediated function.

### 2.5. Regulation of Sp1 Controls TMBIM6 Promoter Activity and mRNA Expression

**Figure 5:**
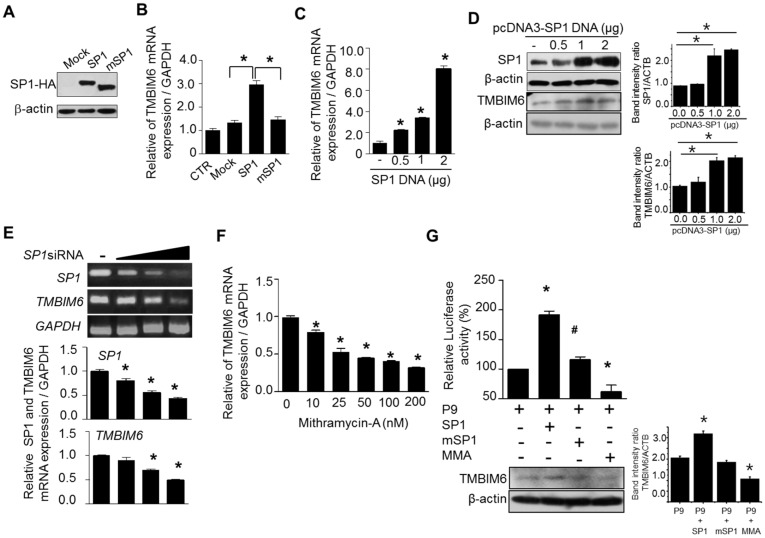
Sp1 activity and expression level are associated with human TMBIM6 expression pattern. (**A**) Western blot showing the overexpression of HA tagged Sp1 and deletion mutant of Sp1. (**B**) The effect of wild type and mutant Sp1 overexpression on TMBIM6 mRNA expression in HT1080 cells, measured by qRT-PCR. The empty vector pCMV-3T3A was used as the vector control (V-CTR); CTR, control non-transfected cells. (**C**) qRT-PCR analysis of TMBIM6 mRNA levels in HT1080 cells transfected with different concentration of wild type Sp1 construct for 24 h. (**D**) Western blot showing the Sp1 and TMBIM6 expression levels after transfecting wild type Sp1 in HT1080 cells for 24 h, respective band intensity ratio with β-actin. (**E**) (upper panel) RT-PCR data showing the Sp1 and TMBIM6 mRNA level after transfection of different concentrations of Sp1-siRNA for 24 h; (lower panel) the respective data of qRT-PCR analysis of Sp1 and TMBIM6 mRNA levels in HT1080 cells. (**F**) qRT-PCR analysis of TMBIM6 mRNA levels in HT1080 cells after treatment with different concentrations of the Sp1 transcriptional activity inhibitor mithramycin-A for 24 h. (**G**) The pGL3-P9 construct of TMBIM6 was transiently transfected into HT1080 cells with either wildtype or mutant Sp1 at the ratio of 2:1 for 24 h, and then the cells were lysed and the luciferase activity was measured. The mithramycin-A effect on Sp1-induced luciferase activity with Sp1 overexpression was also tested at the concentration of 200 nM. The pGL3-basic vector was used as the control. The upper panel shows the promoter activity, while the lower panel shows the western blot for TMBIM6 expression and respective band intensity ratio with β-actin under same conditions. The results are the mean ± SD from *n* = 3 independent experiments. ‘*’ indicate significant differences from the control. ‘#’ indicates the significant difference of P9 activity between Sp1 and mSp1 transfected cells.

In our experiments, we found that the Sp1 transcription factor is critically involved in the promoter activity of TMBIM6. To understand whether inhibition or overexpression of Sp1 affect the promoter activity, we co-transfected wild type Sp1 or mutant Sp1 expression plasmids with minimal promoter region P9 of TMBIM6 in HT1080 cells (Figure 5A). We also treated some cells with mithramycin-A (MMA), an inhibitor of Sp1 binding to DNA. Furthermore, Sp1-dependent endogenous TMBIM6 mRNA expression was analyzed either by overexpression or siRNA-mediated downregulation of Sp1 in HT1080 cells. Overexpression of wildtype Sp1 increased TMBIM6 expression 3-fold (Figure 5B) and we also observed that both TMBIM6 mRNA and protein expression were increased in Sp1 concentration dependent manner (Figure 5C,D). Downregulation of Sp1 with Sp1 specific siRNA (#6667-1, Bioneer Inc, Daejeon, Korea) or inhibition of Sp1 binding to DNA by MMA in HT1080 cells reduced the TMBIM6 mRNA expression significantly in a dose dependent manner (Figure 5E,F). Additionally, the measurement of luciferase activity and TMBIM6 protein expression support that the overexpression of wild type Sp1 increased the promoter activity of TMBIM6 2-fold, and application of MMA inhibited that induction (Figure 5G). These observations suggest that regulating Sp1 expression alone can positively or negatively affect TMBIM6 expression.

### 2.6. Sp1-Dependent TMBIM6 Expression Protects against Stress

To understand the importance of Sp1-mediated TMBIM6 high expression in cancer, P9-TMBIM6-HA or P9-(mSp1d, mSp1p)-TMBIM6-HA constructs were generated and expression levels of TMBIM6 was evaluated. Sp1 site mutant promoter construct P9-(mSp1d, mSp1p)-TMBIM6-HA showed significantly reduced TMBIM6-HA expression than P9-TMBIM6-HA transfected cells (Supplementary Figure S6A), indicating the requirement of Sp1 site for the TMBIM6 expression. Later, different carcinoma cell lines such as MDA-MB 231, MCF7, DU145, and HT1080 were transfected with P9-TMBIM6-HA or P9-(mSp1d, mSp1p)-TMBIM6-HA and treated with anticancer agents paclitaxel and cyclophosphamide at different concentration for 24 h, then cell viability was assessed. Results showed higher viable cells in P9-TMBIM6-HA transfected cells compared to P9-(mSp1d, mSp1p)-TMBIM6-HA cells (Supplementary Figure S6B,C), indicating the Sp1-dependent TMBIM6 expression role in the cancer cell protection against stress. In addition, the TCGA dataset analysis was carried out to analyze the impact of high expression of Sp1 and TMBIM6 on the prognosis of breast invasive carcinoma patients. The analysis showed that the risk of breast cancer mortality increased with high expression of Sp1 and TMBIM6 (Supplementary Figure S7A,B). Furthermore, RNA-seq data analysis from TCGA data showed a positive correlation between TMBIM6 and Sp1 expression in breast cancer patient’s sample (Supplementary Figure S7C).

### 2.7. PMA Enhances Endogenous TMBIM6 mRNA Expression

**Figure 6:**
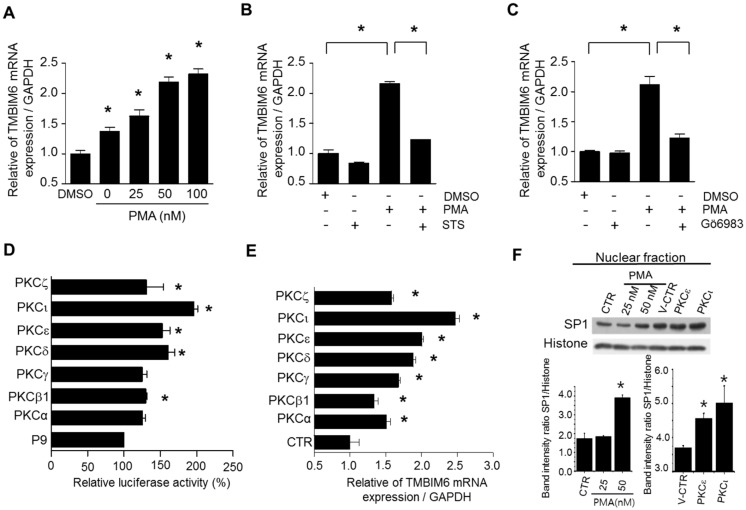
PMA and active PKC isoforms enhances human TMBIM6 gene expression. (**A**) Relative endogenous TMBIM6 mRNA levels after treating HT1080 cells with different concentrations of PMA for 6 h. DMSO was used as the vehicle control. (**B**,**C**) qRT-PCR was performed in HT1080 cells with PKC inhibitors as staurosporine (**B**) and Gö6983 (**C**). HT1080 cells were treated with 100 nM staurosporine (STS) and Gö6983 for 60 min, and then PMA was added, and the cells were incubated for a further 3 h. The results are the mean ± SD from *n* = 3 independent experiments. ‘*’ indicate significant differences from the control. (**D**,**E**) Luciferase assay (**D**) and qRT-PCR (**E**) were performed in HT1080 cells with transiently overexpressing PKC isoform constructs. HT1080 cells were seeded, transfected with pGL3-TMBIM6-P9, and empty pcDNA3.1 vector or with the indicated catalytically active PKC constructs and β-gal plasmid for 24 h. Luciferase activity is presented as percentage induction by considering pGL3- TMBIM6-P9 activity with the empty pcDNA3.1 vector as 100%. (**F**) Western blots show the Sp1 levels in the nuclear fractions of HT1080 cells treated with PMA for 3 h or transfected with PKCε and PKCι for 12 h, histone was used as loading control, lower panel shows the band intensity ratio of Sp1 to histone. The results are the mean ± SD from *n* = 3 independent experiments. Asterisks indicate significant differences from the control.

As explained in the introduction, the expression of BI-1 is enhanced in many cancers. To understand the possible stimulatory mechanisms of TMBIM6, we performed gene induction analyses. In general, tumors have unique environmental conditions, such as hypoxia, inflammation, ER-stress, and a high cell proliferation rate. Therefore, to find a model agonist, we tested several chemical agents that might influence TMBIM6 gene expression, such as hypoxia inducers (cobalt chloride (COCl_2_), deferoxamine), ER stress-inducing agent (thapsigargin), an inflammation-inducing agent (arachidonic acid), protein kinase-A activators (dBcAMP, forskolin), and a protein kinase-C activator (PMA) in HT1080 cells. We treated HT1080 cells with different concentrations of each agent for 6 h and then observed the TMBIM6 mRNA level using qRT-PCR. Most of the treatments did not enhance TMBIM6 expression (Supplementary Figure S8). However, the protein kinase-C pathway activator PMA consistently enhanced TMBIM6 gene expression by 2- to 2.5-fold, with the maximum at 100 nM (Figure 6A). To validate that the PMA-mediated increase in TMBIM6 expression occurred through PKC activation, we evaluated TMBIM6 mRNA expression in the presence of the pan-PKC inhibitors staurosporine and Gö6983 (Figure 6B,C). Both inhibitors significantly blocked the PMA-mediated expression increase. These observations suggest that the activation of PKC family proteins enhances TMBIM6 gene transcription.

### 2.8. PKCι, PKCε, and PKCδ Isoforms Enhance TMBIM6 Gene Expression

The PKC family contains many isoforms, each of which has an individual role in the cell system. There are three groups of isoforms: conventional PKCs (α, β1, β2, γ), novel PKCs (δ, θ, μ, ε, η), and atypical PKCs (ι, ζ). Therefore, to identify which PKC isoform(s) enhance basal TMBIM6 mRNA expression, we transiently expressed catalytically active mutants of some of the isoforms and then tested the luciferase activity and endogenous TMBIM6 mRNA expression in HT1080 cells. To test their effects on promoter activity, the P9 promoter was co-transfected with the individual PKC isoforms, along with a β-gal plasmid. After 24 h, cells were lysed, and the luciferase activity was measured. All PKC isoforms enhanced the promoter activity of P9, but only PKCδ, PKCε, and PKCι caused significant enhancement, by 60%, 55%, and 80%, respectively (Figure 6D). The effect on endogenous mRNA was tested 24 h after the transfection of active PKC mutants, with similar results. PKCδ and PKCε enhanced the expression 2-fold, and PKCι enhanced the expression >2.5-fold (Figure 6E). It is surprising that PKCι showed the maximum enhancement of both promoter activity and mRNA expression because it is insensitive to PMA treatment. Expression and activity of PKC isoforms were confirmed by analyzing PKC regulated genes expression such as cyclin D1 [23], cIAP [24], and claudin [25] (Supplementary Figure S9). To further understand the mechanism of PKC mediated TMBIM6 expression, we analyzed the Sp1 levels in the nuclear fractions of PMA treated and PKCε and PKCι transfected cells. Results showed the increased levels of Sp1 in 50 nM PMA treated and PKC isoforms transfected cells (Figure 6F). Furthermore, immunofluorescence data confirmed the high nuclear Sp1 in PMA-treated and PKCι-transfected cells (Supplementary Figure S10). In addition, the PKC and Sp1-mediated TMBIM6 expression was confirmed by treating PMA in Sp1 knock down cells. HT1080 cells transfected with Sp1 siRNA for 24 h, following by PMA (50 nM) treatment showed no induction of TMBIM6 as compared to scrambled siRNA treated cells (Supplementary Figure S11). Altogether, these results thus infer that the PKC-induced enhanced TMBIM6 expression is mediated through Sp1, and possibly increasing the nuclear Sp1.

### 2.9. Expression Levels of PKCι, Sp1, and TMBIM6 in Human Breast, Prostate, and Liver Cancers

**Figure 7:**
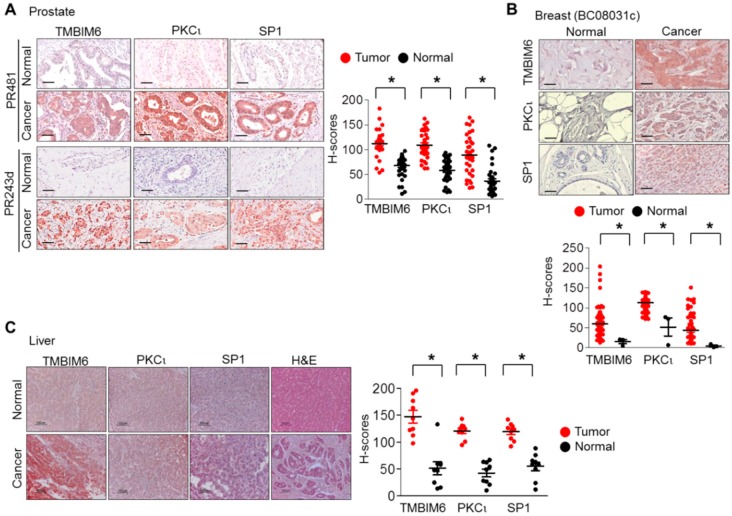
TMBIM6, Sp1, and PKCι expression increased in cancer patient samples. (**A**–**C**) Immunohistochemical staining of prostate (**A**), breast (**B**) tissue microarray sections and liver samples (**C**). Lower and right panel were presents of quantification data. Scale bar represents 100 μm. (brown: positive antibody staining, blue: hematoxylin for nucleus staining). ‘*’ indicate significant differences of respective gene expression between normal and tumor tissue.

To confirm the Sp1 and PKC-mediated enhanced expression of TMBIM6, tissue sections of human breast, prostate, and liver cancers were procured and performed an immunohistochemistry analysis using PKCι-, Sp1-, and TMBIM6-specific antibodies. We have analyzed tumor tissue arrays of prostate and breast samples, and nine samples from liver cancer patients. In H-score analysis, we found significantly increased expression levels of Sp1, PKCι, and TMBIM6 proteins in cancer tissues from normal tissues (Figure 7). These data suggest that Sp1 and PKCι possibly play a role in the enhanced expression of human TMBIM6 in cancers.

## 3. Discussion

In this study, we offer the first report of the basal transcriptional regulation of the human TMBIM6 gene and the possible mechanisms related to its enhanced transcription in cancer. We have identified the core promoter region of the human TMBIM6 gene, transcriptional variant-1, and demonstrated its functional activity. Furthermore, we identified potential transcriptional factor binding sites and their importance in the basal regulation of TMBIM6 using a site-directed mutation study.

Reporter gene functional analyses of progressively truncated 5′-flanking region fragments of the TMBIM6 gene carried out in carcinoma cell lines with different tissue origins (HT1080, MCF-7, DU145, and HepG2) demonstrated similar activity, suggesting that the basal transcription of the TMBIM6 gene is regulated similarly regardless of the cell type (Figure 1B). All six constructs, including the smallest construct (P6, −430/+30), showed activity similar to the longest construct, P1. Therefore, the P6 construct is sufficient to drive the basal transcription of the TMBIM6 gene. However, its activity might be enhanced by distal elements depending on tissue or disease factors. The P2 construct (Figure 2) showed the maximal functional activity, but the P2 specific region (−2170/−1440) did not show that level of activity, and P2 without the P6 region (−430/+30) lost activity altogether (Figure 2F,G), indicating that the P2 region might contain upstream transcription enhancer elements. The upstream enhancer elements in long-range promoters might have a role in tissue-specific expression or certain pathological conditions [26]. However, any distant enhancer elements in the P2-specific region remain to be elucidated. 

To identify the minimal or core promoter region required for basal activity, the P6 construct was fragmented and cloned to a reporter gene (Figure 2F). Those constructs demonstrated that the P9 region (−133 to +30 bp) has a functional level similar to that of the P1 and P6 constructs (Figure 2G). Therefore, P9 contains the core or essential promoter region of TMBIM6 and is sufficient to drive gene expression.

Computer-based analyses revealed that TMBIM6 is a TATA-box-less promoter and that the core promoter region possesses two evolutionarily conserved Sp1 sites (GC box), one each of ETS-1 sites, Elk-1, NF-Y (CAAT box), and NF-kB sites. All these transcription factors commonly found in TATA-box-less promoters and also well evolutionally conserved among several species. To understand the functional relevance of the binding sites for those transcription factors on the TMBIM6 promoter, we performed site-directed mutagenesis studies. The TMBIM6 core promoter has two Sp1 sites; to test their functional relevance, they were mutated individually and together in the P9 construct. Individual mutation of either Sp1 site reduced the functional activity by 65% (Figure 3B), whereas double mutation reduced the activity by 90% (Figure 4B). In some cases, Sp1 driven gene regulation is influenced by other co-transcription factors such as NF-Y and ETS-1 [27,28], this could be the reason that proximal Sp1 site mutation with other sites significantly affected the TMBIM6 promoter activity (Figure 3B). Our results also showed that ETS-1 site mutation resulted in more activity reduction compared NF-kB, NF-Y, and ElK-1 sites, as ETS-1 site is overlapped with Sp1 proximal site, mutation of ETS-1 might partially affected the Sp1 activity. In previous studies, the cooperation of Sp1 and ETS-1 in gene regulation was observed [29,30]. Therefore, ETS-1 role can be expected in the Sp1-mediated TMBIM6 expression. Sp1 is known to regulate TATA-box-less promoters [31,32,33] and also involved in the basal transcription of many other antiapoptotic genes, such as survivin, neurogenin, PADI6, and LRP5 [34,35,36,37]. Disruption of either Sp1 binding site resulted in a significant reduction in promoter activity, indicating the requirement of both sites for maximal activity of TMBIM6 promoter. These results are consistent with previous studies in which a single Sp1 site mutation reduced the basal activity by 60% [34,35]. Therefore, it is possible that Sp1 is involved in the basal transcription of TMBIM6 gene but we were unable to specify which of the two sites is more important in the transactivation of TMBIM6 expression. Nonetheless, the proximal Sp1 site is conserved among rats, mice, and humans (Supplementary Figure S4), suggesting its importance and cooperation from the distal site might be required to achieve full activation.

A previous study [38] reported that increased Krüppel-like zinc finger transcription factor 10 (Klf10) suppressed TMBIM6 expression in an estrogen-dependent manner and induced cell death in adenocarcinoma cells. The Klf10 transcription factor is known for its competitive binding to Sp1 sites (GC-box) and inhibits Sp1 binding, silencing of Sp1-mediated transcription [39,40]. These observations clearly support our finding that the basal transcription of TMBIM6 is mediated mainly by Sp1.

Furthermore, if Sp1 controls the transcription of TMBIM6, higher Sp1 levels might increase the promoter activity or TMBIM6 mRNA expression. As hypothesized, the plasmid-mediated overexpression of exogenous Sp1 in HT1080 cells enhanced the basal promoter activity and transcription of TMBIM6 in a dose-dependent manner. On the other hand, inhibition of Sp1 with MMA, a drug that interferes with the binding between Sp1 and GC-rich sequences [41], inhibited both the promoter activity and the endogenous mRNA expression of TMBIM6 in HT1080 cells in a dose dependent manner. Similarly, MMA has been reported to inhibit the Sp1-mediated expression of many genes [42,43], suggesting that Sp1 levels or the association between Sp1 and specific genomic elements affect the endogenous expression of TMBIM6.

In this study, we found that Sp1-dependent TMBIM6 overexpressing cells are less sensitive against paclitaxel and cyclophosphamide treatment (Supplementary Figure S6), indicating a role of Sp1-dependent TMBIM6 expression in the cancer cells resistance to stress. As endoplasmic reticulum (ER) stress plays an important role in cancer development and chemotherapy resistance [44,45], anticancer agents paclitaxel and cyclophosphamide could induce ER stress-mediated cell death [46,47,48]. The TMBIM6 is well known for the inhibition of ER stress-mediated cell death [4]. Therefore, it is possible that high expression of TMBIM6 could protect against cell death induced by paclitaxel or cyclophosphamide. Supporting this observation, the TCGA dataset analysis (Supplementary Figure S7) showed that the breast cancer patients with high expression levels of Sp1 and TMBIM6 increased the risk of cancer mortality. In addition, the correlation analysis of RNA-seq data of 422 breast cancer samples in TCGA database infers that Sp1 could activate the TMBIM6 expression.

Additionally, we also worked to identify the possible mechanisms involved in the enhanced TMBIM6 gene expression found in cancers by analyzing various chemical inducers. We found that PMA increases the endogenous TMBIM6 level by >2.0-fold in HT1080 cells. PMA is an analogue of DAG, which is known to activate the PKC signaling pathways, which regulate the expression of several genes, primarily those involved in cell proliferation, apoptosis, differentiation [49], and tumorigenesis [50]. Therefore, PMA can be implicated in a variety of PKC-mediated cellular functions in addition to gene expression regulation [51,52,53]. PMA has been reported to induce the expression of various genes by stimulating multiple transcription factors, primarily Sp1, AP1, NF-κB, and AP2 [54,55,56]. In this study, we found that PMA increased the TMBIM6 mRNA level in HT1080 cells. PMA-mediated enhancement of TMBIM6 gene induction was further confirmed by using the broad PKC inhibitors staurosporine and Gö6983. Due to the lack of information about the specific isoforms involved in TMBIM6 enhancement, we used broad spectrum inhibitors to confirm the signaling pathway involved in PMA-induced, PKC-mediated TMBIM6 upregulation. The TMBIM6 upregulation in the PMA treatment condition could be mediated also through Sp1. Sp1 was previously shown to mediate the PMA-induced expression of membrane-bound GC-A in human monocytic THP-1 cells and the expression of the TMBIM6 gene in airway epithelial cells [57,58]. In this study, NF-κB and AP2 sites were found in the core promoter region of TMBIM6; therefore, the PMA mediated-enhancement of TMBIM6 expression might also be mediated through those factors [56]. However, in Sp1 knockdown cells the PMA treatment did not induce the TMBIM6 expression. It has been reported that alterations in the Sp1 phosphorylation levels affect its DNA-binding activity [59]. PMA treatment can induce the Sp1 phosphorylation and its DNA-binding activity [58]. Therefore, the PMA-mediated enhanced TMBIM6 gene expression is affected during Sp1 knockdown conditions. However, future studies on detailed mechanisms of PMA-mediated TMBIM6 gene induction might give more insight of TMBIM6 gene expression regulation. On the other hand, chemical-induced ER stress, hypoxia, and inflammation did not alter the level of TMBIM6 mRNA (Supplementary Figure S8).

In trying to confirm the PKC-mediated enhanced expression of the TMBIM6 gene by overexpressing catalytically active mutants of PKC isoforms, we found that PKCδ, PKCε, and PKCι significantly enhanced both the core promoter region activity and the basal expression of TMBIM6 mRNA (Figure 6D,E). Those results corroborate the observation of PMA-enhanced TMBIM6 transcription because all PKC isoforms, except the atypical PKCι and PKCζ, isoforms are known to be activated by PMA. Interestingly, PKCι is the most potent isoform with respect to the induction of both promoter activity and mRNA expression of the TMBIM6 gene. PKCδ reportedly has both proapoptotic and antiapoptotic functions depending on the cell type [60]. In various cancer types—such as breast cancer, prostate cancer, and human malignant fibrous histiocytoma—PKCδ is highly expressed and behaves as a pro-survival factor [61,62,63]. Inactivation of PKCδ resulted in reduced proliferation and survival of cancer stem cells [64]. PKCδ supports the survival of MDA-MB-231 breast cancer cells and non-small cell lung cancer (NSCLC) cells [65,66], in which TMBIM6 expression is reportingly increased. Furthermore, PKCδ and PKCε are also highly expressed in cancer types such as breast and prostate, and mainly upregulates NF-κB mediated transcriptional signaling [61,67,68]. PKCε mediates PMA-induced mucin gene expression in human colonic cell lines [69].

Additionally, PKCι has been reported as a tumorigenic protein kinase in various cancers. In breast cancers, PKCι is the atypical-PKC isoform most commonly overexpressed [70,71]. Our data indicates that Sp1 plays a very important role in TMBIM6 gene transcription. The phosphorylated state of Sp1 is known to exert positive influence on transcriptional function by increasing the stability and DNA binding activity [58,72,73,74]. Therefore, the kinases such as PKC could influence the Sp1-mediated transcriptional activity by increasing Sp1 nuclear stability and DNA binding activity. Phosphorylation of Sp1 sequential to the O-glycosylation increases the nuclear compartmentalization [75]. Interestingly, atypical-PKC isoform-mediated phosphorylation of Sp1 positively regulates the Sp1-mediated genes expression in cholangiocarcinoma, which resulted in enhanced invasion and metastasis by influencing growth factor expression and tumor angiogenesis [76,77]. PKCα regulates the transcription of Nix and Bnip3 in Sp1 dependent manner [78]. Furthermore, it was reported that PKC-mediated Sp1 activation was involved in the upregulation of cell proliferation through the induction of IGF-II gene activation [79]. All of this evidence supports our observation of higher levels of nuclear Sp1 in PMA treated and PKCε and PKCι overexpressing cells, leading to the possible enhanced induction of TMBIM6 gene in cancer by Sp1 in PKC dependent manner.

The altered expression of PKC isoforms in several tumors could directly or indirectly affect gene expression and could thus be one possible reason that TMBIM6 expression is significantly enhanced in tumors. Moreover, our data (Figure 7) are consistent with those in previous studies [69,70,80], where PKCι, PKCε, and PKCδ were shown to significantly induce gene expression in different cancers. Additionally, the immunohistochemistry data demonstrated the expression pattern of the PKCι, Sp1, and TMBIM6 is well matched in breast, prostate, and liver cancer clinical tissue samples. As mentioned above, most of the PKC isoforms are also highly expressed in various cancers. Therefore, involvement of PKC isoforms, other than PKCι, in the TMBIM6 expression cannot be undermined. The high expression character of other TMBIM family proteins has also been observed in many cancers. Members of the TMBIM family such as TMBIM1, TMBIM2, TMBIM4, and TMBIM5 are found to be expressed highly in various cancers and also play an important role in cell adhesion and migration [81,82,83,84]. Additionally, TMBIM3 found to be involved in the gastric cancer progression by modulating the glycolytic metabolism [85]. These observations corroborate the high expression of TMBIM6 and its importance in cancer.

## 4. Materials and Methods

### 4.1. Reagents and Expression Plasmids

Deferoxamine, Forskolin, 8-bromoadenosine 3′,5′-cyclic monophosphate (cAMP), phorbol 12-myristate 13-acetate (PMA), COCl_2_, thapsigargin (Tg), and staurosporine were purchased from Sigma-Aldrich (St. Louis, MO, USA). Gö6983 was obtained from Abcam (Cambridge, UK), and mithramycin-A was purchased from Cayman Chemicals (Ann Arbor, MI, USA). Expression plasmids of wild type Sp1 and functionally inactive mutant Sp1, as previously reported [86], were purchased from Addgene, Watertown, MA, USA, (#12098 and #12097, respectively) and sub-cloned to pcDNA3 vector. Catalytically active mutant isoforms plasmids of PKC family were kindly donated by Professor, Jae-Won Soh (Inha University, Incheon, Korea). Details of the PKC mutant isoforms were previously published [23].

### 4.2. Cell Culture

HT1080, HeLa, MCF7, HepG2, MDA-MB-231, and DU145 cells purchased from the Korean Cell Line Bank (Seoul, Korea). The cells were cultured in DMEM complete (Gibco BRL, Grand Island, NY, USA) with 10% fetal bovine serum (FBS; Gibco BRL), 100 U/mL penicillin, and 100 μg/mL streptomycin (Invitrogen, Waltham, MA, USA). The cells were cultured at 37 °C in a humidified atmosphere with 5% CO_2_.

### 4.3. Quantitative Real-Time PCR (qRT-PCR)

To quantify the relative expression of TMBIM6 after treatment with different chemical compounds, we performed real-time PCR. The mRNA expression levels were determined by qRT-PCR as previously described [87]. Total RNA was isolated from cells with TRIzol reagent (Invitrogen Life Technologies, Carlsbad, CA, USA), and complementary DNA was synthesized from RNA using a PrimeScript Reverse Transcriptase kit (TaKaRa, Shiga, Japan) according to the manufacturer’s protocol. Quantitative real-time PCR was performed using SYBR Green PCR Master Mix (Applied Biosystems, Foster City, CA, USA) according to the protocols provided by the manufacturer and using an ABI prism 7700 Sequence Detector System (Applied Biosystems). The real-time PCR program consisted of an initial denaturation at 95 °C for 10 min followed by 40 cycles of 95 °C for 15 s; 60 °C for 20 s. The human TMBIM6 primers used in this study are listed in Table 1. Glyceraldehyde 3-phosphate dehydrogenase (GAPDH) mRNA expression was used as an endogenous control.

### 4.4. Transcription Start Site Determination by 5′-RACE-PCR

The 5′ cDNA end was amplified using the GeneRacer kit (Invitrogen) according to the manufacturer’s instructions. In brief, total RNA was isolated from a fibrosarcoma cell line (HT1080) and breast adenocarcinoma cell line (MCF7) as explained above. 2 μg of the total RNA was used to generate cDNA with a 5′-cap using the kit according to the manufacturer’s instructions. The synthesized cDNA containing a potential full-length 5′ end was used as a template in nested PCR. The primers used for the first PCR were GeneRacer 5′ primer (5′-CGACTGGAGCACGAGGACACTGA-3′) and a gene-specific primer (5′- GGCAGGGCCCAGGCCAACTCCTGTA-3′) located in exon 2 of the TMBIM6 gene. The PCR amplifications were carried out in 25 μL volumes containing one unit of TaqDNA polymerase (Invitrogen). The PCR conditions were 94 °C for 2 min, 94 °C for 30 s, and 72 °C for 1 min for 5 cycles; 94 °C for 30 s and 72 °C for 1 min for 5 cycles; 94 °C for 30 s, 65 °C for 30 s, and 72 °C for 1 min for 30 cycles; and 72 °C for 5 min using an automated thermal cycler (GenAmp^®^PCR System 9700, PE Applied Bio Systems, Foster city, CA, USA). The PCR product was separated in a 2% gel. Then 1/100 diluted PCR product was re-amplified using a nested primer pair: GeneRacer 5′ nested primer (5′-GGACACTGACATGGACTGAAGGAGTA-3′), provided by the company, and a gene-specific primer (5′-CCAAGGCAGACAGCAGGCCAGCCT-3′) for exon 2 of TMBIM6. The nested PCR product was separated in a 2.5% gel. The DNA in the gel was isolated using a kit (QBiogene, Carlsbad, CA, USA) and cloned into a TA cloning pGEMT easy vector (Promega, Madison, WI, USA). Plasmid DNAs were purified using a commercial kit (Promega) and sequenced at Bioneer Biotechnology Company (www.bioneer.com).

### 4.5. Isolation of the 5′ Flanking Region/Promoter of the Human TMBIM6 *Gene*

Human genomic DNA was extracted from the HT1080 cell line using the phenol/chloroform extraction protocol. According to the available sequence for human *TMBIM6* genomic DNA (Gene code:ENSG00000139644.12, Gene transcript ID:ENST00000267115.9/GenBank accession NM_003217.3) in the UCSC genome browser gateway (https://genome-asia.ucsc.edu/), we randomly selected a region 2430 bp upstream from the 5′ untranslated region (5′UTR)/first exon and designed primers with KpnI restriction enzyme sites for the sense primer and BglII for the antisense primer (SP: 5′-ATCGGTACCTAGGTGTACCGGGACTCTAAAAAGAA-3′, ASP: 5′-ACTAGATCTTACCAGCGCTTCTAACACCGGATG-3′). The selected region was amplified from 50 ng of genomic DNA from HT1080 cells by PCR (GenAmp^®^PCR System 9700, PE Applied Bio Systems) using an i-pfu DNA amplification kit (iNtRoN Biotechnology, Inc., Seongnam, Korea) with i-pfu DNA polymerase and following the manufacturer’s protocol. The PCR profile was 5 min at 94 °C for 1 cycle; 20 s at 94 °C, 20 s at 60 °C, and 5 min at 72 °C for 35 cycles; and 5 min at 72 °C. The amplified PCR product was added with single deoxyadenosine to the 3′-ends by incubating it with 1 U of Taq polymerase at 72 °C for 10 min for TA cloning. Then, it was cloned into the pGEM-T Easy vector (Promega) to obtain plasmid pGEM-TMBIM6, which was later subjected to DNA sequencing at Bioneer Biotechnology Company (www.bioneer.com).

### 4.6. Generation of TMBIM6 Promoter-Luciferase Reporter Plasmid Constructs

A series of luciferase reporter gene constructs was prepared in the promoter-less pGL3-basic luciferase reporter gene vector (Promega) to determine the promoter activity of the 5′-upstream region of the human TMBIM6 gene. The promoter region in the pGEM-TMBIM6 construct that contained part of the 5′-UTR and upstream flanking region (−2430/+30 from the transcription start site) was separated by *KpnI* and *BglII* digestion and sub-cloned into the pGL3-basic vector to make the reporter gene construct pGL3-TMBIM6-P1(−2430/+30). Then, five progressive 5′-deletion constructs (pGL3-TMBIM6-P2 to pGL3-TMBIM6-P6) were generated through PCR amplification by using different forward primers with the *KpnI* site and a common reverse primer with the *BglII* restriction enzyme site. Additionally, P2 specific region construct (P2ΔP3) and P2 without P6 (P2ΔP6) construct were prepared from pGL3-TMBIM6-P2 reporter construct by PCR amplification using primers specific for the indicated region. P6 region (−405/+30) was further fragmented into −405/−260, −259/−134, and −133/+30 by PCR amplification and prepared the pGL3-TMBIM6-P7, pGL3-TMBIM6-P8, and pGL3-TMBIM6-P9 respectively by cloning into luciferase reporter pGL3 basic vector. The primer sequence and enzyme sites are listed in the Table 1. The nucleotide sequences of all constructs were verified for orientation.

### 4.7. Luciferase Activity and TMBIM6 mRNA Induction

The different cell lines named above were seeded in a 12-well dish at 1 × 10^5^ cells per well. The following day, 1 μg of each luciferase reporter TMBIM6 promoter construct and 0.1 μg of a pCMV-β-gal plasmid (Clontech, Shiga, Japan) containing a β-galactosidase gene were co-transfected using Lipofectamine™ 3000 (LifeTechnologies, Roche, Indianapolis, IN, USA) according to the manufacturer’s specifications. The pGL3-control vector (Promega, Madison, WI, USA), an internal control plasmid for Renilla luciferase expression, and the promoter-less pGL3 basic vector were used as negative control in all experiments. The cells were lysed with 150 μL of lysis buffer after 24 h of transfection, and the luciferase activity was measured by the Dual-Luciferase Activity Detection System (Promega). Luciferase activity was calculated by normalizing the values with respect to β-galactosidase activity to correct for differences in transfection efficiency. The luminescence of all experiments was determined using Lumat LB 9507 (Berthold Technologies, Bad Wildbad, Germany). The results are presented as the normalized mean values from at least three independent transfections. The reporter activity is expressed in terms of percentage, considering pGL3 basic vector activity value as 1%.

To determine the modulatory effect of PKC isoforms on TMBIM6 core promoter activity, HT1080 cells were seeded at a density of 1 × 10^5^ cells/well in a 12-well plate. For reporter assays, the cells were transfected using Lipofectamine (Invitrogen) with 1 μg of pTMBIM6-P9 reporter plasmid, 500 ng of active catalytic mutants of PKC isoforms, and 15 ng of the transfection control plasmid pCMV-β-gal. The pcDNA3.1 plasmid was used a vector control. Then, 8 h after transfection, fresh media was replaced, and the cells were incubated for a further 24 h. Then, cell extracts were prepared and luciferase assays were done using a luciferase assay system (Promega). To find the effects of Sp1 overexpression, cells were co-transfected with 500 ng of pGL3-TMBIM6-P9 reporter plasmid, 250 ng of expression plasmids of Sp1 and mSp1, and 10 ng of the pCMV-β-gal expression plasmid. After 8 h, fresh media was provided, and the cells were incubated for a further 24 h. Then, cell extracts were prepared for the luciferase assay, which was carried out in triplicate and repeated three times. To determine endogenous mRNA expression modulation, HT1080 cells were seeded at a density of 1 × 10^5^ cells/well in a 12-well plate. 1 μg of active catalytic mutants of PKC isoforms and 1 μg of Sp1 or mSp1 plasmids were transfected individually. After 8 h, fresh media was provided, and after 24 h of incubation, the cells were harvested with Trizol reagent, and total RNA was extracted and processed for qRT-PCR as explained above.

### 4.8. Site-Directed Mutagenesis Analysis

The Muta-Direct^TM^ site-directed mutagenesis kit (iNtRON Biotechnology, Seongnam, Korea) was used according to the manufacturer’s instructions to generate constructs with mutations at different binding elements. The primers used to introduce mutations in the P8 constructs are listed in Table 2. The following PCR conditions were used: 95 °C for 30 s followed by 16 cycles of 95 °C for 30 s, 55 °C for 60 s, and 72 °C for 6 min. The PCR products were digested with Mutazyme enzymes at 37 °C for 1 h and transformed into HIT competent cells-DH5α (Real Biotech Corporation (RBC), Banqiao City, Taiwan). Mutations in double transcription factor-binding sites were generated in single-mutation constructs by repeating primer-directed mutagenesis with different sets of primers. The generated constructs with the mutated sequences were verified using direct DNA sequencing.

### 4.9. Chromatin Immunoprecipitation (ChIP) Assay

ChIP was carried out on HT1080 and MCF7 cells using the Pierce agarose ChIP kit (Thermo Scientific, Rockford, IL, USA) according to the manufacturer’s instructions. Briefly, 2 × 10^6^ cells per reaction were cross-linked by incubation with a final concentration of 1% formaldehyde for 10 min at room temperature. Then the reaction quenched by 1× glycine for 5 min. Subsequently, the cells were washed twice with cold phosphate buffered saline (PBS) and then harvested by scraping in 1 mL cold PBS containing 10 µL of Halt cocktail with a protease inhibitor. After pelleting and resuspension in lysis buffer, the nuclei were digested with micrococcal nuclease for 15 min at 37 °C. The digested chromatin was then incubated with IgG and agarose for 1 h to pre-clear the chromatin. Immunoprecipitations were done by incubation with anti-Sp1 (CST,#9389), anti-ETS-1 (CST,#14069), anti-NF-YA (sc-17753X), and anti-SP4 (sc-390124X) at the supplier’s recommended dilution rate and anti-RNA polymerase II (Pol II) and normal rabbit IgG (kit contents) antibodies overnight at 4 °C on a rocking platform. ChIP-grade agarose beads were then added for one-hour incubation at 4 °C, followed by washing. The crosslink was reversed by heating at 65 °C for 40 min, followed by treatments with RNase A for 30 min at 37 °C and proteinase K for 1.5 h at 65 °C. The DNA was column purified. The PCR reactions were carried out for 30 cycles: 94 °C for 20 s, 58 °C for 20 s, and 72 °C for 30 s. The sequences of primers are listed in Table 1. The expected amplicon was 260 bp, and the PCR product was resolved by gel electrophoresis on 1.5% agarose.

### 4.10. Electrophoretic Mobility Shift (EMSA) and Supershift Assays

HT1080 and MCF-7 (source) cells were cultured in 100 mm plates in DMEM containing 10% FBS and grown for 24 h. Nuclear extracts were prepared using a Nuclear Extraction Kit (Thermo, Rockford, IL, USA). The oligonucleotides corresponding to the two Sp1 sites in the TMBIM6 core promoter region were synthesized and 3′-end biotin labeling was done using a Pierce™ Biotin 3′ End DNA Labeling Kit (Thermo, Rockford, IL, USA) and annealed. The sequence of the biotin-labeled probe corresponding to the Sp1 binding regions is listed in Table 2. Then a LightShift Chemiluminescent EMSA kit (Thermo) was used according to the manufacturer’s protocol. In brief, 20 µL of 1× binding buffer, 50 ng/µL of Poly (dI.dC), 0.05% NP-40, 2 μg of nuclear extracts, 4 pmol of unlabeled competitor oligonucleotides, mutated unlabeled competitor oligonucleotides, and 20 fmol of labeled probe were applied and incubated at room temperature for 20 min. In the supershift assay, 0.2 μg of Sp1 (CST,#9389) antibody was added to the respective reactions and incubated for an additional 20 min at room temperature, normal rabbit IgG was used as a control. Then DNA-protein complexes were loaded onto a 6% non-denaturing polyacrylamide gel for blotting.

### 4.11. DNA Sequence Alignment and Database Analysis of the 5′-Flanking Region of TMBIM6

The genomic sequences and mRNA sequences of TMBIM6 were obtained from GenBank and the UCSC database (https://genome.ucsc.edu/). To identify transcriptional start site location, RACE-PCR product sequences were aligned with the TMBIM6 genomic sequences available in GenBank (NM_003217.3). To find putative transcription factor binding sites, we used the MatInspector professional (http://www.genomatix.de/) and TFSEARCH online programs, considering binding sites or elements with matrix similarity of >0.9 from the core promoter region. Sequence alignment for the genomic sequences of the 5′- flanking region of TMBIM6 from humans, mice, and rats was carried out using the ClustalW2 algorithm (http://www.ebi.ac.uk/Tools/msa/). To confirm the sequence identity with promoter constructs or mutated constructs, we used the Multalin (http://multalin.toulouse.inra.fr/multalin/) program.

### 4.12. Stimulation or Inhibition of Promoter Activity and Endogenous TMBIM6 mRNA Expression

HT1080 cells were seeded at a density of 1 × 10^5^ cells per well in 12-well plate. After overnight incubation, the cells were treated with different stimulators (PMA, forskolin, 8-bromoadenosine 3′,5′-cyclic monophosphate (cAMP), thapsigargin, cobalt chloride (CoCl2), arachidonic acid and deferoxamine) at various concentrations for 6 h. Then the cells were harvested with 500 μL of Trizol reagent to determine mRNA expression by means of qRT-PCR. To determine PMA-induced, PKC-mediated TMBIM6 mRNA expression, the pan-PKC inhibitors staurosporine and Gö6983 were used both individually. For the inhibition study, cells were pre-exposed to PKC inhibitors for 30 min, then treated with PMA for 3 h, harvested with Trizol, and analyzed for mRNA expression. Mithramycin-A treatments were carried out for 24 h. All stimulator and inducer stock solutions were prepared in DMSO. In each experiment, a vehicle control was maintained, and all assays were repeated at least three times.

### 4.13. Western Blotting

In vitro cultured HT1080 cells transfected with HA tagged Sp1 for 24 h, then cells were harvested and homogenized in RIPA buffer containing protease and phosphatase inhibitors for 30 min at 4 °C. After centrifugation at 13500 rcf at 4 °C for 30 min, the supernatants were collected. The protein concentration was measured using the Pierce bicinchoninic acid (BCA) assay kit. The samples were mixed with 4× loading buffer, and equal amounts of protein were loaded into each well of 10% SDS-PAGE gels. Then, the separated proteins were transferred to PVDF membranes (Bio-Rad, Hercules, CA, USA). After being blocked with 5% skimmed milk in TBST buffer for 2 h, the membranes were incubated with primary antibodies at 4 °C overnight and then washed with TBST buffer, 10 min each for 3 times. Then incubated with secondary antibody for 1 h at RT. After being washed with TBST buffer, blots were visualized using an enhanced chemiluminescence (ECL) kit (Merck Millipore, Darmstadt, Germany). Primary antibodies were used at the following dilutions: rabbit anti-Sp1 (1:1000, #9389; CST), rat HA (1:1000, Roche), rabbit anti-TMBIM6 (1:1000, #ab18852, Abcam), and mouse anti-β-actin (1:1000, sc-47778).

### 4.14. Immunohistochemistry

Human paraffin embedded tissue microarrays of breast and prostate cancer were procured from US Biomax, Inc., Rockville, MD, USA) (catalog #PR481, PR243d for prostate cancer, catalog #BC08013c for breast cancer). Human liver biospecimens were provided by the Chonbuk University Hospital Biobank, all samples were obtained with informed consent under institutional review board-approved protocols (Jeonju, Korea, 2011-08-012). Subjected to 1× Target Retrieval Solution, pH 6.0 (DAKO, Glostrup, Denmark). Sections were incubated with a peroxidase blocking solution (DAKO) for 10 min at room temperature. They were then washed with 1× TBST buffer (Scytek Lab, Logan, UT, USA) followed by a protein block (0.25% casein in PBS, DAKO) for 10 min at room temperature. Primary antibodies, anti-PKCι (1:100, anti-rabbit, Abcam, Cambridge, UK), anti-TMBIM6 (1:100, anti-rabbit, Abcam, Cambridge, UK), and anti-Sp1 (1:1000, anti-rabbit, #9389) were diluted in antibody diluent provided by DAKO and incubated in a humidified chamber overnight at 4 °C. Slides containing tissue sections were further rinsed in TBST buffer and incubated with the indicated secondary antibodies for 1 h at room temperature. AEC substrate chromogen (DAKO) was added, and then the cells were washed with deionized water. This was followed by counter staining with Mayer’s hematoxylin (Sigma-Aldrich). The slides were rinsed with tap water and mounted using an aqueous medium (Scytek Lab, West Logan, UT, USA). H-scores analysis were performed using Fiji ImagJ software (National Institute of Health (NIH), Bethesda, MD, USA).

### 4.15. Cell Viability Assay

To analyze the Sp1 mediated TMBIM6 expression effect on the cell viability during stress, we have generated the constructs by subcloning ORF of TMBIM6-HA from pCDNA3-TMBIM6-HA plasmid into P9 promoter construct and named as P9-TMBIM6-HA, in which P9 promoter drives the TMBIM6-HA expression. Another constructs was generated by subcloning ORF of TMBIM6-HA into Sp1 sites mutated P9 promoter construct and named as mP9-TMBIM6-HA. Cell lines representing different cancer were seeded into 96-well plates at the density of 1 × 10^4^ cells/well. After 12 h of seeding cells were transfected with P9-TMBIM6-HA or mP9-TMBIM6-HA plasmid. After 24 h of transfection cells were treated with different anticancer drugs paclitaxel and cyclophshomide at different concentrations in triplicate for 24 h. After 24 h of treatment, cell viability was assessed using the cell counting kit-8 (Enzo life science, Farmingdale, NY, USA, #ALX-850-039-KI01) by following the manufacturer’s protocol. The data represented is the percentage of viable cells in a well and were normalized to the untreated control wells for each agent.

### 4.16. TCGA Data Acquisition and Analysis

The gene expression data for breast invasive carcinoma [88] were downloaded from TCGA. Overall survival analysis of breast cancer in TCGA datasets was performed using a web tool OncoLnc (http://www.oncolnc.org) [89]. RNA-Seq data obtained from TCGA database and evaluated for the correlation of Sp1 and TMBIM6 expression.

### 4.17. Statistical Analysis

All statistical analyses were performed using Origin Pro 8 Version 8.0951 (Northampton, MA, USA) and GraphPad (GraphPad Software, Inc., San Diego, CA, USA) statistical software. One-way analysis of variance (ANOVA) with Tukey post hoc test and Student’s *t*-test were performed. Significance was set at a *p*-value of * < 0.05. All observations are expressed as the mean ± SD. In each case, the statistical test used is indicated, and the number of experiments is stated in the legend of each figure.

## 5. Conclusions

In this study, we have identified the minimal/core promoter of the human TMBIM6 gene and concluded that two Sp1 binding sites between positions -72 and -53 relative to the TSS are critical to its basal transcription. Inhibition of the DNA binding of Sp1 or overexpression of Sp1 regulated the promoter activity and endogenous TMBIM6 expression. RNA-seq analysis of breast cancer patient’s samples from the TCGA database inferred the positive correlation between Sp1 and TMBIM6 gene expressions. Here, we reported for the first time on the PKC activation-mediated enhanced basal transcription of TMBIM6. Among the activated PKCs, PKCι showed the maximum influence on human TMBIM6 gene expression. These data provide information about the basal transcriptional regulation of the TMBIM6 gene and also hint at the reasons behind the increased expression of TMBIM6 in cancers. This report can assist in further investigations into the regulation of the human TMBIM6 gene and could help find novel intracellular signaling pathways to treat TMBIM6-associated diseases.

## Figures and Tables

**Table 1:** Primers used for the reporter construct and quantitative real-time PCR (qRT-PCR)

**Construct**	**Forward (5′→3′)**	**Reverse (5′→3′)**
pGL3-TMBIM6-P1	atctggtaccgggactctaaaaag (*KpnI*)	atcagatcttaccagcgcttctaacacc (*BglII*)
pGL3-TMBIM6-P2	atcggtaccaaaatgaatgtggatactggtct (*KpnI*)	atcagatcttaccagcgcttctaacacc (*BglII*)
pGL3-TMBIM6-P3	atcggtacctcatattgtcttatgcccaatttc (*KpnI*)	atcagatcttaccagcgcttctaacacc (*BglII*)
pGL3-TMBIM6-P4	atcggtacctcctgctacactacagcaaaaa (*KpnI*)	atcagatcttaccagcgcttctaacacc (*BglII*)
pGL3-TMBIM6-P5	atcggtaccgcctactgagaatcataccctg (*KpnI*)	atcagatcttaccagcgcttctaacacc (*BglII*)
pGL3-TMBIM6-P6	atcggtaccttttaatgtgttctctgaatcg (*KpnI*)	atcagatcttaccagcgcttctaacacc (*BglII*)
pGL3-TMBIM6-P2ΔP3	atcggtaccaaaatgaatgtggatactggtct (*KpnI*)	atcaagcttggggtggtctcctcccttatt (*HindIII*)
pGL3-TMBIM6-P2ΔP6	atcggtaccaaaatgaatgtggatactggtct (*KpnI*)	atcagatctaaaaaaaaaaagcgccctcgcaag (*BglII*)
pGL3-TMBIM6-P7	atcggtaccttttaatgtgttctctgaatcg (*KpnI*)	atcagatctctctagtggagaaagtgaaaatg (*BglII*)
pGL3-TMBIM6-P8	atcggtaccgcagcagcgcaccgtgac (*KpnI*)	atcagatctataaacagcggctgcttag (*BglII*)
pGL3-TMBIM6-P9	atcggtaccgcacgtacagccaatgg (*KpnI*)	atcagatcttaccagcgcttctaacacc (*BglII*)
**qRT-PCR**		
*hTMBIM6*-v1	gagcacatccggtgttagaa	gtgttgccatcagccaaatc
*GAPDH*	ctcagacaccatggggaaggtg	ctcagccttgacggtgccatg

Note: V1-variant 1, Δ-deletion.

**Table 2:** Primers/probes used for site-directed mutations and the EMSA and ChIP assays.

**Primers Used for Site-Directed Mutations**	
**Name**	**Sequence 5′→3′**
mSp1-d-S	aagcggaccacaggTATgggtttccctcgagaggc
mSp1-d-AS	gcctctcgagggaaacccATAcctgtggtccgctt
mSp1-p-S	tcgggagcggaagtggTTAagtcagagcacatcc
mSp1-p-AS	ggatgtgctctgactTAAccacttccgctcccga
mNFY-p-S	gtgcggccaacggcTTTtgaggactggtcttaag
mNFY-p-AS	cttaagaccagtcctcaAAAgccgttggccgcac
mNFkB-S	accacagggcggggtCATcctcgagaggcgaacg
mNFkB-AS	cgttcgcctctcgaggATGaccccgccctgtggt
mElk-1-S	cgagtcagagcacatAAgTtgttagaagcgctggt
mElk-1-AS	accagcgcttctaacaAcTTatgtgctctgactcg
mETS1-S	gtaaaagatcgggagcTAaagtgggcgagtcagag
mETS1-AS	ctctgactcgcccacttTAgctcccgatcttttac
**Probes Used in EMSA**	
**Name**	**Sequence 5′ to 3′**
Sp1-1-S	gcggaccacagggcggggtttccctcgagaggc
Sp1-1-AS	gcctctcgagggaaaccccgccctgtggtccgc
Sp1-2-S	cgggagcggaagtgggcgagtcagagcacatccg
Sp1-2-AS	cggatgtgctctgactcgcccacttccgctcccg
**Note:** Mutant primers mentioned above are used as probes for respective sites	
**ChIP Assay Primers**	
G-TMBIM6 S	cgacaagctcaggcctctgaac
G-TMBIM6 AS	taccagcgcttctaacaccgga

Indication: m—mutation; S—sense; AS—antisense; G—genome.

## Supplementary Materials

The following are available online at https://www.mdpi.com/2072-6694/11/7/974/s1, Figure S1: TMBIM6 expression pattern, Figure S2: Sequence of 5′-flanking region of the human TMBIM6 gene selected for the promoter assay, Figure S3: Putative transcription factor binding sites present in the P1 specific region (−2460/−2171) and P2 specific region (−2171/−1439) of the TMBIM6 promoter region, Figure S4: The human TMBIM6 core promoter P9 sequence (−133/+30) was aligned with the same upstream length as the transcription site or 5′ cDNA end sequence of mouse and rat TMBIM6 available in the NCBI database or UCSC, Figure S5: EMSA assay showing distal Sp1 site binding to the human TMBIM6 promoter, Figure S6: Sp1-dependent TMBIM6 expression protects against stress, Figure S7: Overall survival analysis of breast cancer patients and RNA-Seq analysis, Figure S8: Induction of endogenous TMBIM6 mRNA expression, Figure S9: Expression and activity analysis of catalytically active-mutants of PKC isoforms, Figure S10: HT1080 cells transfected with indicated PKC isoforms for 12 h or treated with PMA for 3 h, Figure S11: PMA did not induce the TMBIM6 mRNA expressions in Sp1 knock down conditions.

## Abbreviations

C/EBP, CCAAT enhancer binding protein; ChIP, Chromatin immune precipitation; Elk-1, ETS Like-1 protein; EMSA, Electrophoretic mobility shift assay; hTMBIM6, Human Transmembrane Bax Inhibitor-1 Motif-containing 6; NF-Y, Nuclear transcription factor Y; NFκB, Nuclear factor kappa B; Sp1, Specificity protein 1; PKC, Protein Kinase-C; PMA, Phorbol 12-myristate 13-acetate; TSS, Transcription start site.
