# Supplementary material for: Expression of TMBIM6 in Cancers: The Involvement of Sp1 and PKC
Source: Cancers (Basel). 2019 Jul 11;11(7):974. doi: 10.3390/cancers11070974 (PMC6678130; doi:10.3390/cancers11070974)
Supplement: Supplementary file 1 [file cancers-11-00974-s001.pdf]

Supplementary Materials

Expression of TMBIM6 in Cancers: The Involvement of Sp1 and PKC

Raghu Patil Junjappa, Hyun-Kyoung Kim, Seong Yeol Park, Kashi Raj Bhattarai, Kyung-Woon Kim, Jae-Won Soh, Hyung-Ryong Kim and Han-Jung Chae

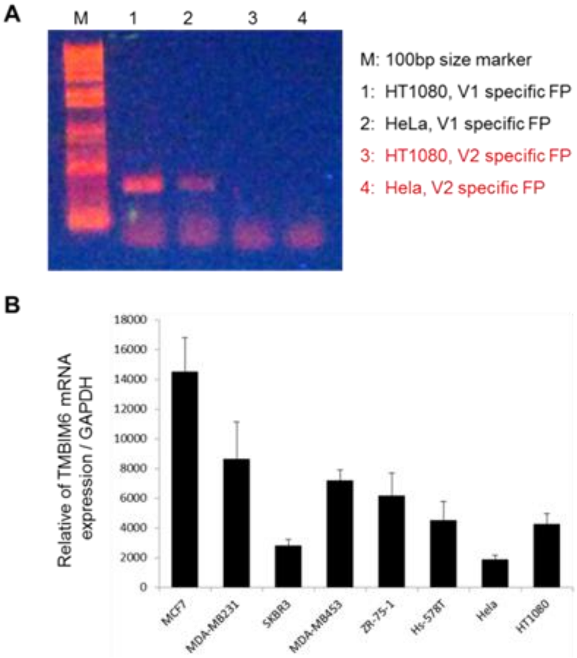

Figure S1. TMBIM6 expression pattern. (**A**) Analysis of the expression pattern of human TMBIM6 transcriptional variants 1 and 2 in HT1080 and HeLa cells. PCR was done with the 5′ UTR-specific forward primer of each variant with a common reverse primer. (**B**) TMBIM6 mRNA expression pattern in various cancer cell lines.

```
-2430 TAGGTGTACCGGGACTCTAAAAAGAACTTGTTTGTATAATGCTATTCTATACAAGGTATGTAGCCCAGGAA
-2359 ATGACCAACCTGATGTGTGTTATGACCCATCTGAGCCTCCCATGACCACAGTTTTTAAAATAAGATTAAGG
-2288 ACTGAGGACTGGTGGGGAGGACTCATAAATGATACGAGTAAAGTGTTAGCCAAAACAAAAGAAAAA
-2222 GGGGTGCCCAAACAAGTCACCTTGAAGTTTGATGCCTGTGCTGTCATTAATAGCAATAAGTTAGGAAGGG
-2152 GATGTGGCTCTTTTAGTTGGGAAAAAGGCTATATGACCGAAAATAAGTACATTTGTCATGAATGAGGACTG
-2081 TGTGAAAATGAATGTGGATACTGGTCTTGTGTCATTTAGGCCACTTGGATAAAAAATGAAAAGGATCCAGT
-2010 CCACCTTCAGAAAGGAAAAAATGGCTCTTCCTGTACTAAGGGACAATGTAACCCCTTAGAGCTAGTAATA
-1940 ACCAATCCCCTTGATCCTCGCTGGAAAAAAAGGGGAGCGTGTGACCTTAGGAATCGACAGGGCTGGAC
-1872 TGGATCGTCGAGTAAATATCTTAGTTCGAGGAGAAGTCTGTGGAAAATTTAACCTTACCAATTGCTGCCTGCA
-1799 CATAGATGATCAGAGGCAAGTAGTCGAAGATATAGTTAAAGACATGACGAAACTGGCACATGTGCCTGT
-1730 ACAAGTGTGACACGGATTTGACCCTGAAGCTATGTTTAAAAATGGTTCCCAGTGCTAGGAGGATTTAAAAC
-1659 TCTTATAATAAAAATTATAGTAGTAATAGCCTGCTTACTGATCCCTTATTTACTATCTGTACTCATTCAAATGGTAA
-1582 AAGGTTTCATCACTACTCTAGTTCACCAGAATGCTTCAGCACAAGTGTACTACATAAATCACTATCGATCTGT
-1509 CATGCAGGAAGACATAGGTAGTGAAGATGAAGGTGAGAACTCCCACTAATAAAATGAGTGAGAGTCT
-1442 CAAAGTGGGGGAATAAGGGAGGAGACCACCCCTCATATTGTCTTATGCCCAATTTCTGCCTCCAAAGAA
-1373 AGAAGTAGTAAAAACTAAAAGGCAGAAATGAAATCCACAAGCAGACAGCCAGGCACCACACCCT
-1309 GGGCCTCGTAGTTAAAGATCGACCCCTGACCTAATCGGTTATGTTATCTATAGATTACAGACATTGTATAGA
-1237 AAAGCACTGTGAAAATCCCTGTCCTGTTCTGATCTAATTACCGGTGCATGCTTGCTCAATCGATCACTACCCT
-1164 CTCAAGCGGATCCCTTTAGAGTTGTGAGGCCTTAAAAGGGACAGGAATTGCTCACTCAGGGAGCTCAGC
-1095 TCTTGAGACAGGAGTCTTGGCCGATGCTCCTGGCGGAATAAACCGCTTCCTGCTTTAACTCGGTGTCTGAG
-1024 GGGTTTTGTCTGCGGCTTGTCCTGCTACACTACAGCAAAAATTAGGTTGTATTATAGAAAATTAACTTTTTT
-974 GGTTTTTTGAGACGGGGTCTCGCTCTGTCTCCCAGGCTGGAGTGCAGTGGCACCATCTCGGCTCACTGCAACC
-878 TTCACCTCCAGGGTCAAGCAATTCTCCTGCCTCAGCCTCCCGAGTAGTTGGGATTACAGGCACACGCCA
-809 CCACGCCCAGCTAATTTTTTAAATTTTTAATAGAGATGGGGTTTTACCATGTTAGCCAGGCTGGTCTCAAAC
-737 TCCTGGCCTCAAGTGATCTGCCCACCTCAGCCTCCCAAAGTGCTGGGATTACAGACGTAAGCCACCAC
-669 GCCCAGCCGAAAATTAACTTCTATTAATTGCATACCTGGGTTTGCCTACTGAGAATCATACCCTGGATATT
-598 TGTATTCTTTACTGAAAAGAACTGTTCTATCTTTCCAGAAAGTGGTTTCCTAAATTCTGGTTTTCCTCCTGGAAT
-523 CCTGCTATTTCCTTCTCGCATACTCTAGAGAAATCGAAGTGTTTTCAAAGGTGCTCTGAAGCTTACGCTCCA
-451 CTAGTTTCACAGTAAATCGCCCCTTGCGAGGGCGCTTTTTTTTTTTTTTTAATGTGTTCTCTGAATCGCTTTCC
-377 CCCTTTAACACTGCATGACAAGACTAAAGCCAGAATCCCAAGACTGCAGCGCTACCCCTTCACAAGCCG
-308 TCGGGCTGTCCCGGGCGGGGCCGGCCCATTTTCACTTTCTCCACTAGAGGCAGCAGCGCACCGTGAC
-241 CCTGGCTTACTACGGTAAGAAAGCGGAGACCTTTTGCTTCGAGGCGGTTGGTGGCCAGTAGTCTTGATC
-172 GACAAGCTCAGGCCTCTGAACTAAGCAGCCGCTGTTTATGCACGTACAGCCAATGGAAAGGCAGG
-107 AGTGCGGCCAACGGCCAATGAGGACTGGTCTTAAGCGGACCACAGGGCGGGGTTTCCCTCGAGA
-43 GGCGAACGCCGGGTAAAAGATCGGGAGCGGAAGTGGGCGAGTCAGAGCACATCCGGTGTTAGA
                                              +1
      AGCGCTGGTA
              +30
```

Figure S2. Sequence of 5′-flanking region of the human TMBIM6 gene selected for the promoter assay. The pGL3-P1 full length construct was generated with this sequence.

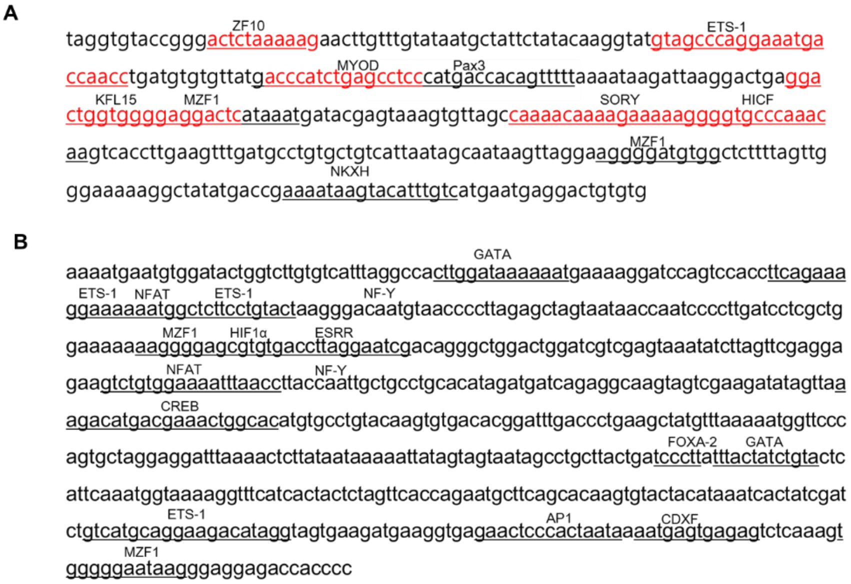

Figure S3. Putative transcription factor binding sites present in the P1 specific region (−2460/−2171) (**A**) and P2 specific region (−2171/−1439) (**B**) of the TMBIM6 promoter region.

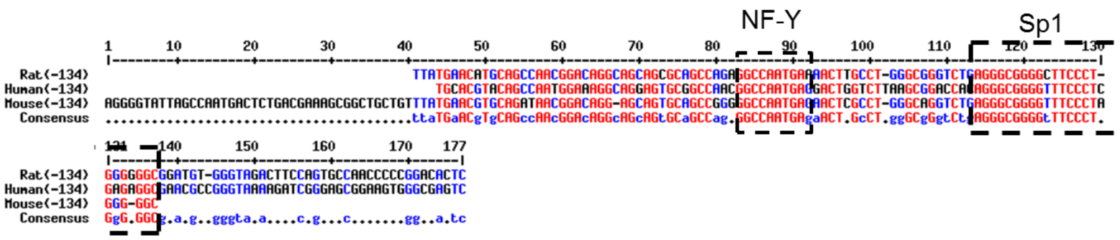

Figure S4. The human TMBIM6 core promoter P9 sequence (−133/+30) was aligned with the same upstream length as the transcription site or 5′ cDNA end sequence of mouse and rat TMBIM6 available in the NCBI database or UCSC. Demarcated site shows the conserved CAAT box and proximal GC box (Sp1 binding site).

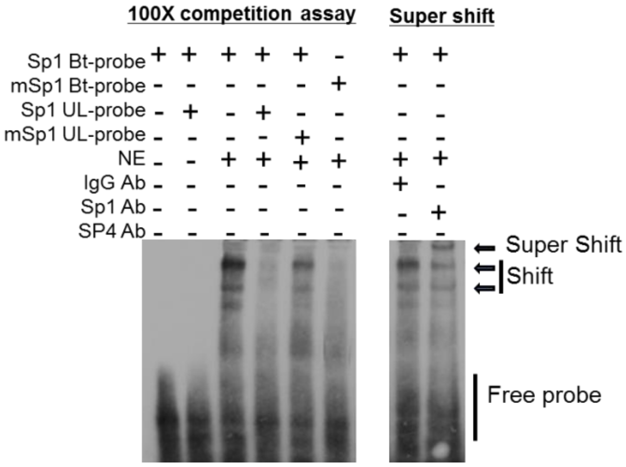

Figure S5. EMSA assay showing distal Sp1 site binding to the human TMBIM6 promoter. Nuclear extract was prepared from HT1080 cells. Double-stranded 35-bp oligonucleotide probe specific to distal Sp1 site (−103/−70) generated and labeled with biotin. The labeled Sp1-p probe was incubated alone (lane 1), with unlabeled probe (lane 2), with the NE (lane 3), with the NE and 200-fold excess amount of unlabeled homologous probe (lane 4), with 200-fold excess mutant probe (lane 5), and with mutant-labeled probe that failed to form a complex with the NE (lane 6). DNA–protein complexes that formed are indicated (S). In lanes 7–9, the super shift assay was performed by pre-incubating the NE with probe for 20 min on ice before adding the specific antibody. Then, the specific probe was added and further incubated for 20 min on ice, with normal IgG as the negative control in lane 7 and anti-Sp1 monoclonal antibody in lanes 8, respectively. The super shift is indicated as SS.

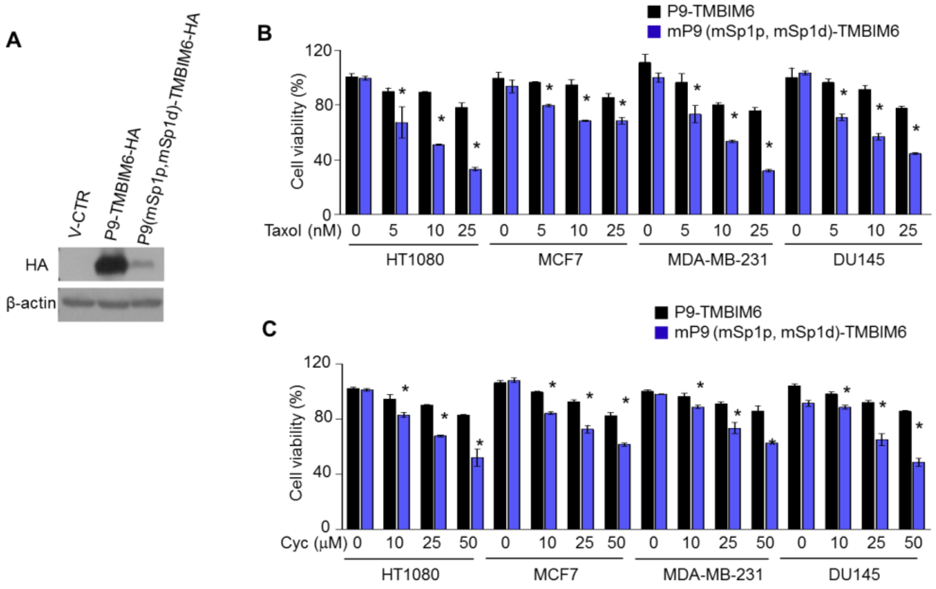

Figure S6. Sp1- dependent TMBIM6 expression protects against stress. (**A**) Immunoblot showing the Sp1 dependent TMBIM6-HA expression in P9-TMBIM6-HA, P9(mSp1p, mSp1d)-TMBIM6-HA and empty vector transfected HT1080 cells. (**B**,**C**) Cell viability percentage comparing to untreated cells, Indicated cell lines were treated with different concentration of paclitaxel and cyclophosphamide for 24 h, and cell viability was assed. The results are the mean ± SD from $n = 3$ independent experiments.

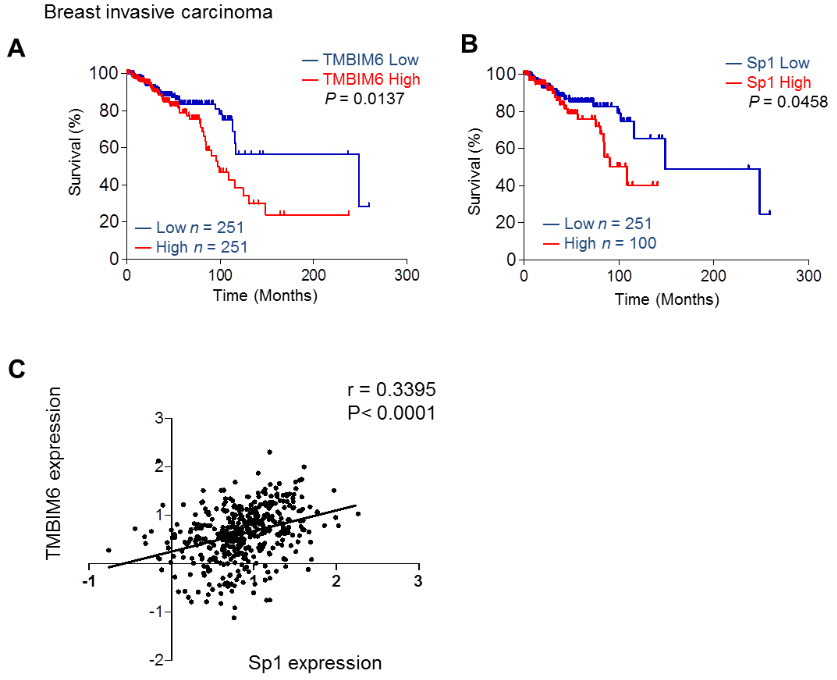

Figure S7. Overall survival analysis of breast cancer patients and RNA-Seq analysis. (**A**,**B**) Kaplan-Meier curves showing the overall survival analysis in patients with high and low expression of TMBIM6 and Sp1 using OncoLnc from TCGA database. p value with log-rank analysis. (**C**) Pearson's correlation coefficient revealed a positive correlation between TMBIM6 and Sp1 expression in breast cancer patient sample from RNA-seq data, TCGA database.

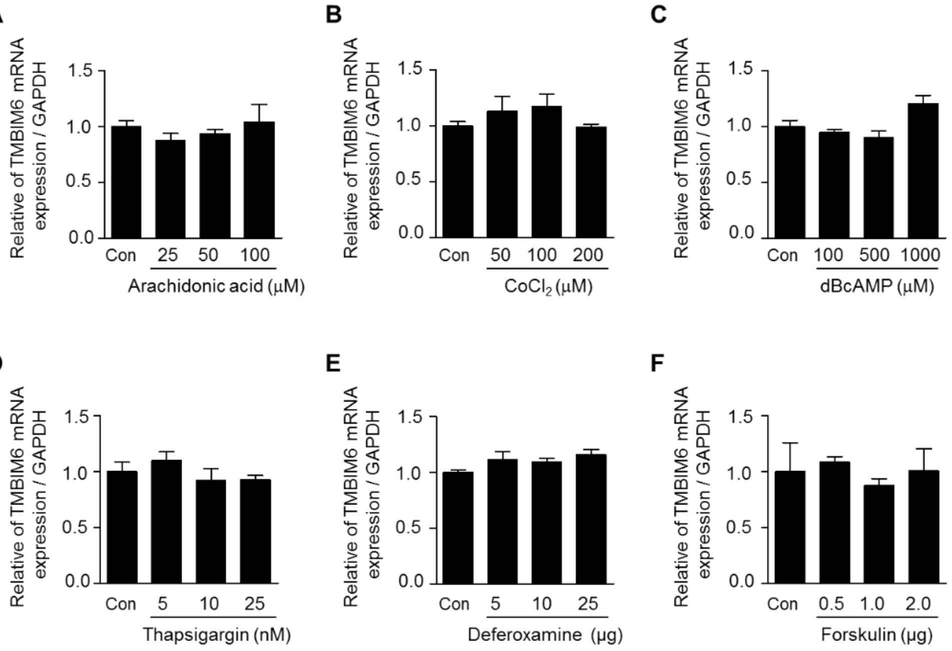

Figure S8. Induction of endogenous TMBIM6 mRNA expression. HT1080 cells were treated with various chemical inducers at different concentrations for 6 h, and the mRNA level was quantified by qRT-PCR. Relative TMBIM6 mRNA expression level was measured in HT1080 cells treated with arachidonic acid (**A**), cobalt chloride ($CoCl_2$) (**B**), dB cyclic AMP (**C**), thapsigargin (**D**), deferoxamine (**E**) and forskulin (**F**) at indicated concentration. Error bars, mean ± SD.

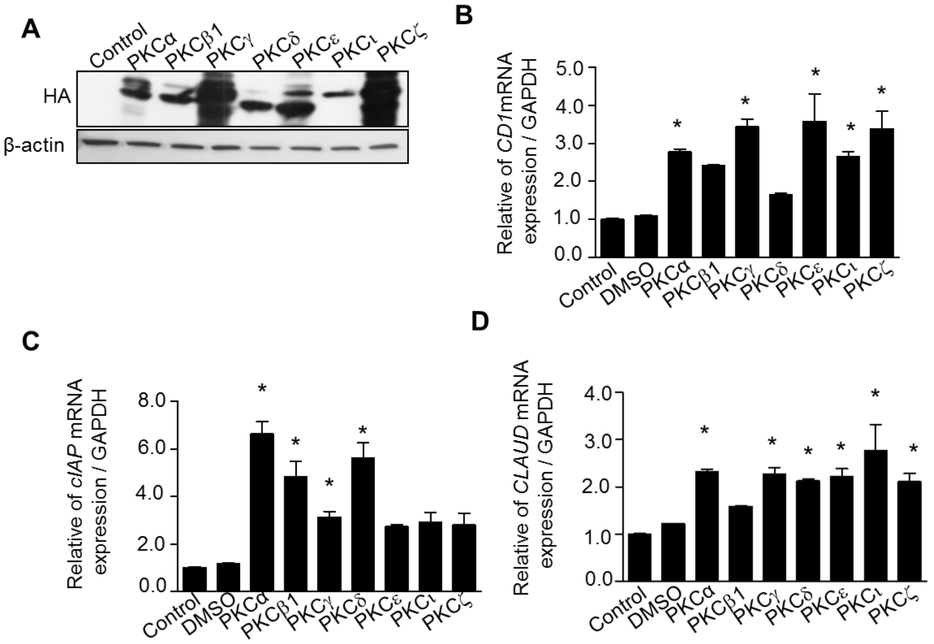

Figure S9. Expression and activity analysis of catalytically active-mutants of PKC isoforms. (**A**)Western blot showing the expression of catalytically active mutants of PKC isoforms, HA tagged mutant isoforms were transfected to HT1080 cells, after 24 h, cells were harvested and analysed for the HA expression. (**B**–**D**) Relative mRNA expressions of CD1, cIAP and CLAUDIN as positive controls for the PKC isoforms activity analysis. '*' indicate significant differences from the control.

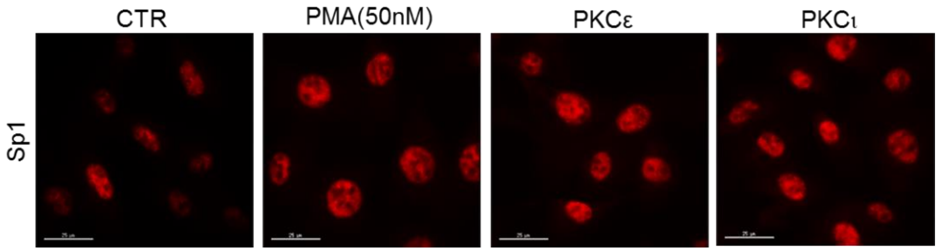

Figure S10. HT1080 cells transfected with indicated PKC isoforms for 12 h or treated with PMA for 3 h. Then cells were fixed and immunofluorescence was done with Sp1 antibody. Magnification: 60×.

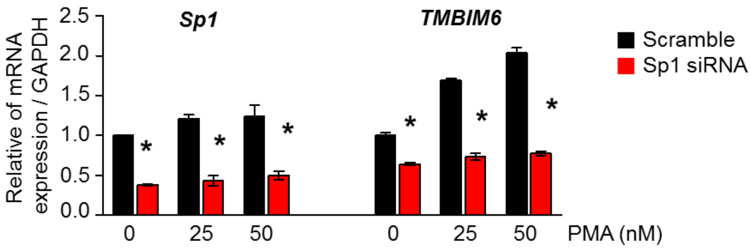

Figure S11. PMA did not induce the TMBIM6 mRNA expressions in Sp1 knock down conditions. HT1080 cells were transfected with Sp1 siRNA and scramble RNA for 24 h. After 24 h, PMA treatment was done 3 h. Cells were harvested in trizol reagent and analyzed for mRNA expressions. The results are the mean ± SD from n = 3 independent experiments. '*' indicate significant differences from the scramble siRNA transfected cells in respective conditions.

Raw Data for Western Blotting

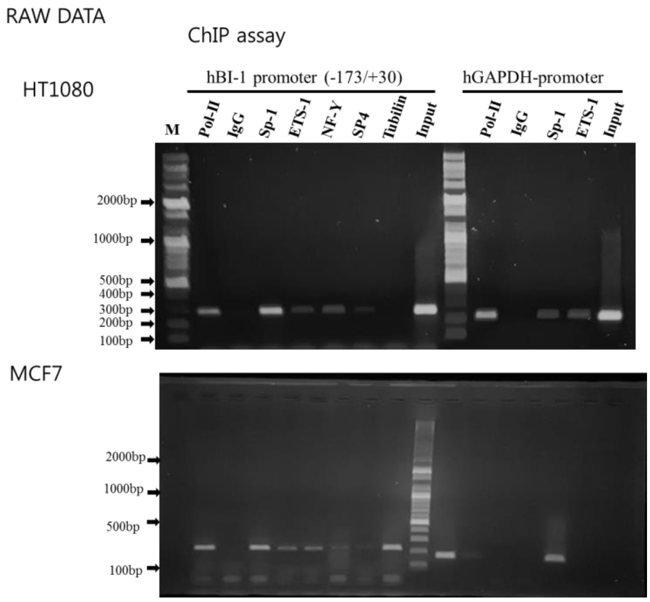

Supplementary: RAW DATA

EMSA:

Figure 4A: HT1080 nuclear extract

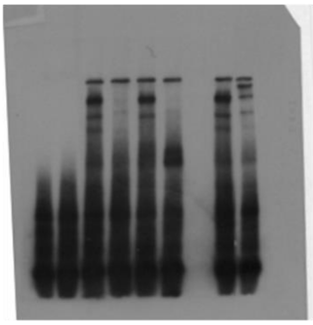

Figure 4B: MCF7 nuclear extract

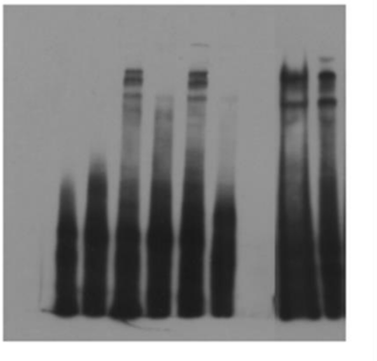

Supplementary figure 4: HT1080 nuclear extract with distal SP1 probes

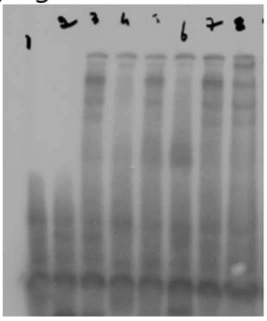

Western blots: Raw data

Figure: 5A

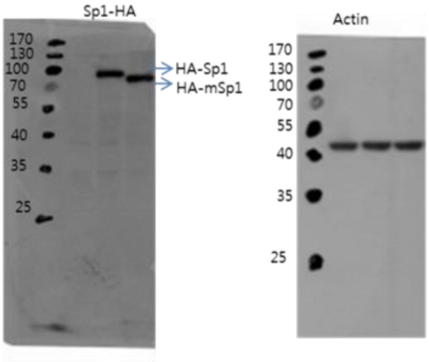

Figure: 5D

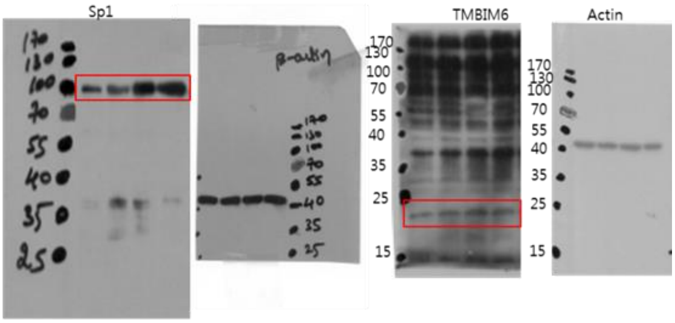

Figure: 5G

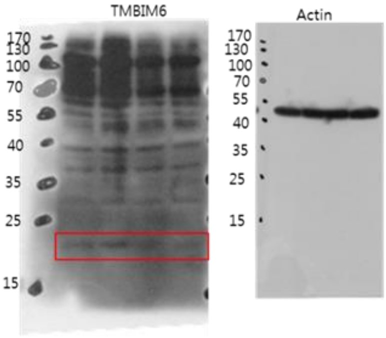

Figure: 6F

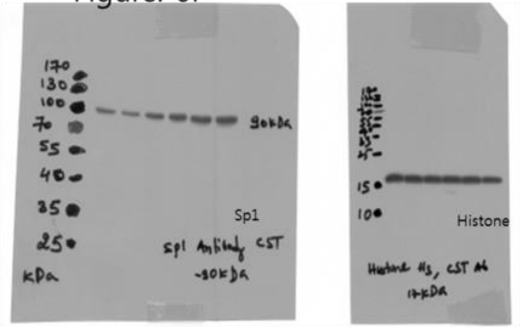

Raw data; RT-PCR for SP1, TMBIM6 and GAPDH

Figure:5E

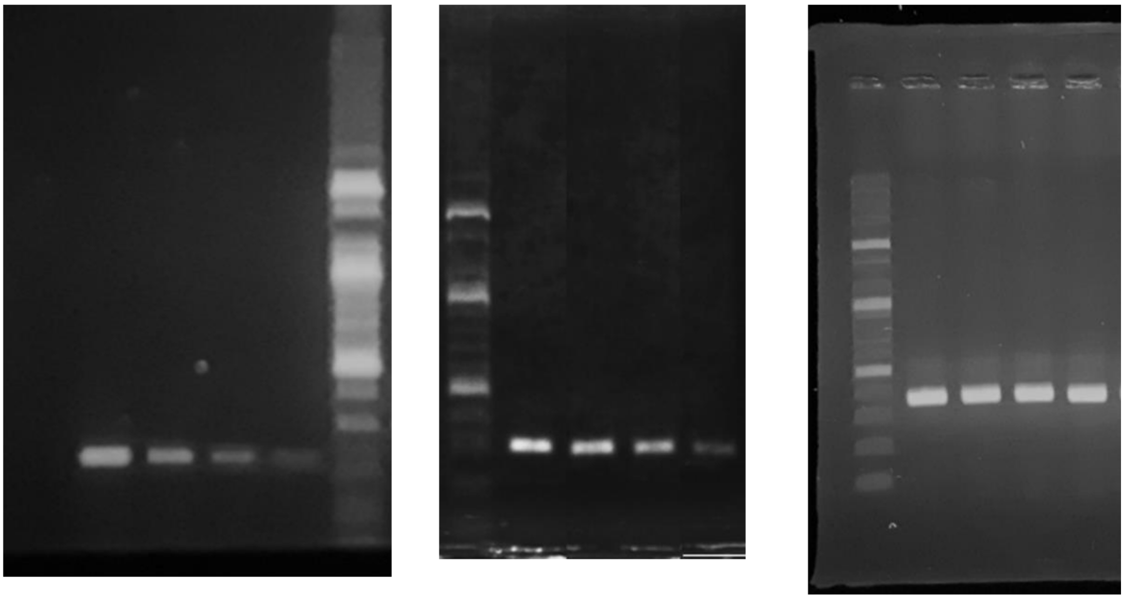

© 2019 by the authors. Licensee MDPI, Basel, Switzerland. This article is an open access article distributed under the terms and conditions of the Creative Commons Attribution (CC BY) license (http://creativecommons.org/licenses/by/4.0/).
